# The bZIP Transcription Factor HapX Is Post-Translationally Regulated to Control Iron Homeostasis in *Aspergillus fumigatus*

**DOI:** 10.3390/ijms22147739

**Published:** 2021-07-20

**Authors:** Manuel Sánchez López-Berges, Mareike Thea Scheven, Peter Hortschansky, Matthias Misslinger, Clara Baldin, Fabio Gsaller, Ernst R. Werner, Thomas Krüger, Olaf Kniemeyer, Jakob Weber, Axel A. Brakhage, Hubertus Haas

**Affiliations:** 1 Institute of Molecular Biology, Biocenter, Medical University of Innsbruck, 6020 Innsbruck, Austria; matthias.misslinger@i-med.ac.at (M.M.); clara.baldin@i-med.ac.at (C.B.); fabio.gsaller@i-med.ac.at (F.G.); 2 Department of Molecular and Applied Microbiology, Leibniz Institute for Natural Product Research and Infection Biology (Leibniz-HKI), 07745 Jena, Germany; Mareike.Scheven@web.de (M.T.S.); Peter.Hortschansky@hki-jena.de (P.H.); Thomas.Krueger@hki-jena.de (T.K.); Olaf.Kniemeyer@hki-jena.de (O.K.); jakob.weber.de@gmail.com (J.W.); 3 Institute of Microbiology, Friedrich Schiller University Jena, 07743 Jena, Germany; 4 Division of Biological Chemistry, Biocenter, Innsbruck Medical University, 6020 Innsbruck, Austria; ernst.r.werner@i-med.ac.at

**Keywords:** iron homeostasis, HapX, *Aspergillus fumigatus*, post-translational regulation

## Abstract

The airborne fungus *Aspergillus fumigatus* causes opportunistic infections in humans with high mortality rates in immunocompromised patients. Previous work established that the bZIP transcription factor HapX is essential for virulence via adaptation to iron limitation by repressing iron-consuming pathways and activating iron acquisition mechanisms. Moreover, HapX was shown to be essential for transcriptional activation of vacuolar iron storage and iron-dependent pathways in response to iron availability. Here, we demonstrate that HapX has a very short half-life during iron starvation, which is further decreased in response to iron, while siderophore biosynthetic enzymes are very stable. We identified Fbx22 and SumO as HapX interactors and, in agreement, HapX post-translational modifications including ubiquitination of lysine^161^, sumoylation of lysine^242^ and phosphorylation of threonine^319^. All three modifications were enriched in the immediate adaptation from iron-limiting to iron-replete conditions. Interfering with these post-translational modifications, either by point mutations or by inactivation, of Fbx22 or SumO, altered HapX degradation, heme biosynthesis and iron resistance to different extents. Consistent with the need to precisely regulate HapX protein levels, overexpression of *hapX* caused significant growth defects under iron sufficiency. Taken together, our results indicate that post-translational regulation of HapX is important to control iron homeostasis in *A*. *fumigatus*.

## 1. Introduction

Fungi are an extremely adaptable class of microorganisms mostly comprised of saprophytes thriving on dead organic material. Nevertheless, a relatively small number of fungal species have evolved a parasitic lifestyle associated with the ability to cause disease in both animals and plants. Among them, around 400 fungal species have been reported as pathogens of mammals [1] with *Aspergillus fumigatus* being one of the most common pathogenic molds in humans [2]. In addition to non-invasive forms of aspergillosis, patients with compromised immune system are at high risk of developing invasive aspergillosis. Today, *A. fumigatus* belongs to the most prevalent airborne fungal pathogens of humans with estimated 3,000,000 cases of chronic pulmonary aspergillosis and 250,000 cases of life-threatening invasive aspergillosis worldwide [3,4]. The lack of early and specific diagnosis and limited therapeutic options aggravate this problem, resulting in a case fatality rate of invasive aspergillosis between 30–90% [3,5]. Considering the rapid evolution of pathogenic fungi [6] and the emergence of resistant strains to currently available treatments [7,8], there is an urgent need for the development of new antifungals, a process that requires a comprehensive knowledge of the cellular mechanisms underlying pathogenicity.

Iron is an essential element for almost every organism since it serves as a cofactor of many cellular processes; however, its excess can be highly toxic to the cell by promoting the production of reactive oxygen species [9,10]. Consequently, iron homeostatic mechanisms are indispensable to guarantee the optimal balance of this metal. In order to understand these dynamic processes, and because adapting to iron limitation has been shown to be essential for fungal pathogenicity in both animal and plant hosts [11,12,13,14,15], several genome-wide transcriptional analyses have been conducted in different fungi over the last 10–15 years [11,12,14,16]. Like other filamentous fungi, *A*. *fumigatus* responds to iron limitation by upregulating iron acquisition mechanisms, such as reductive iron assimilation (RIA) and siderophore-mediated iron uptake, and downregulating iron-consuming pathways, including heme biosynthesis and respiration. This adaptation mainly relies on the bZIP transcription factor HapX and, importantly, it is essential for virulence of *A*. *fumigatus* in a murine model of invasive aspergillosis [11]. Moreover, HapX was also shown to be required for the adaptation to iron excess, mainly via activating vacuolar iron deposition [17]. The dual role of HapX is based on its ability to sense iron via interaction with the monothiol glutaredoxin GrxD and [2Fe-2S] cluster binding [18,19]. *hapX* is highly transcribed during iron starvation, while its expression is repressed by iron [11,20].

**Figure 1:**
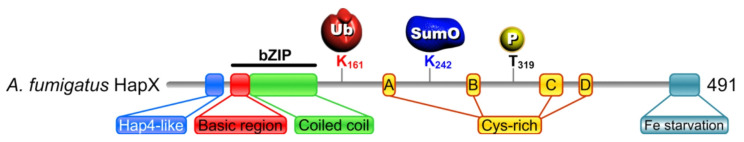
**Schematic representation of the *A. fumigatus* HapX domain organization.** The N-terminal Hap4-like CBC binding domain (CBC-BD) is shown in blue. Basic region and coiled coil domains of the bZIP domain are depicted in red and green, respectively. Cysteine-rich regions are marked in yellow. Cysteine-rich regions A and B are essential for HapX function during iron excess but not during iron starvation, while the C-terminus, shown in turquoise, is crucial during iron starvation. Post-translational modifications of residues K161 (ubiquitination), K242 (sumoylation) and T319 (phosphorylation), objects of this study, are shown in dark red, blue and yellow, respectively.

At the mechanistic level, HapX functions via physical interaction with the CCAAT-binding complex (CBC) [20,21,22] and contains the following domains (Figure 1): A “b(ZIP)” basic and a “coiled-coil” domain mediating DNA-binding, an N-terminal Hap4-like domain mediating interaction with the CBC, and four cysteine rich regions (CRR A-D), which are involved in iron sensing [17]. However, to date, it is unknown whether HapX is additionally regulated at the protein level to control iron homeostasis in *A*. *fumigatus*.

In the current study, we characterized HapX protein regulation during the short-term adaptation from iron-limiting to iron-replete conditions, showing that HapX is quickly degraded throughout this fast adaptation. We identified relevant HapX interactors, such as Fbx22 and SumO, and accordingly, several HapX post-translational modifications, like ubiquitination of lysine 161, sumoylation of lysine 242 and phosphorylation of threonine 319. Disturbing these post-translational modifications affected HapX degradation, heme biosynthesis and iron resistance to different extents. Additionally, in agreement with the necessity to regulate HapX protein levels, overexpression of *hapX* produced significant growth defects under iron sufficiency. Collectively, our results demonstrate that post-translational modification of HapX is important for the maintenance of iron homeostasis in *A*. *fumigatus*.

## 2. Results

### 2.1. HapX Is Rapidly Degraded during Adaptation from Iron-Depleted to Iron-Replete Conditions in a Proteasome Dependent Manner

**Figure 2:**
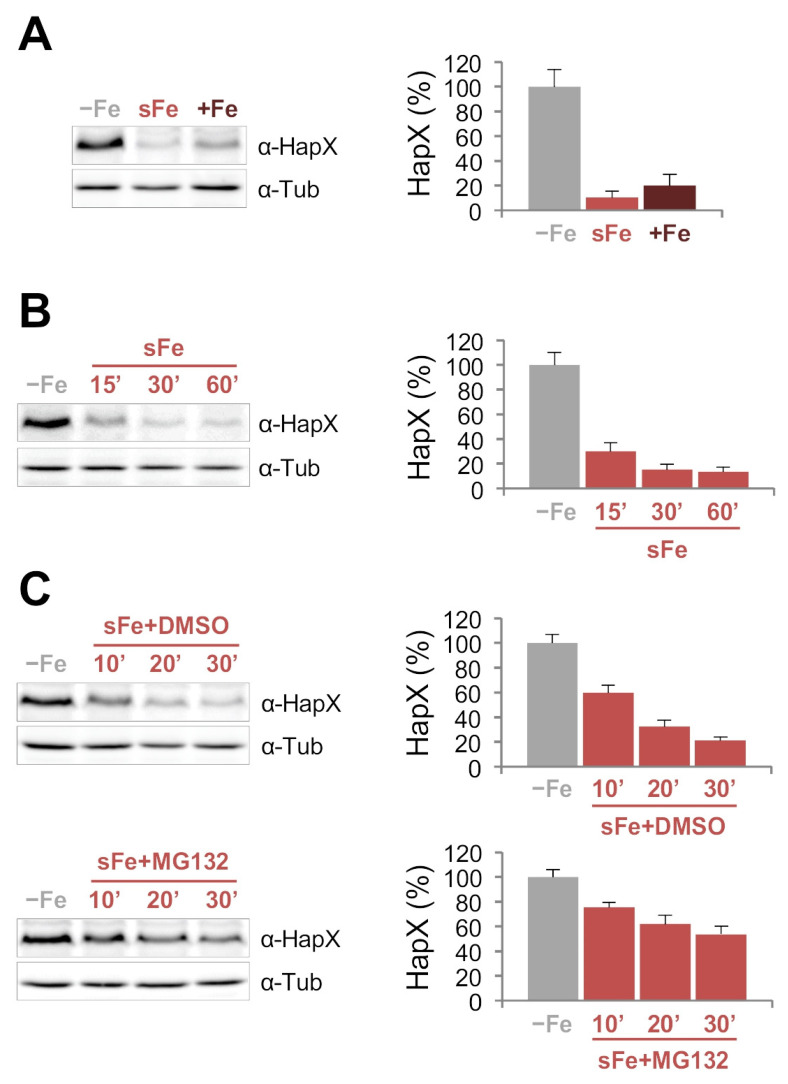
**HapX is rapidly degraded during the adaptation from iron-depleted to iron-replete conditions in a proteasome dependent manner**. (**A**–**C**). HapX quantification by Western blot analysis. Samples were obtained from the wild-type strain grown at 37 °C in liquid Aspergillus Minimal Medium (AMM) −Fe (20 h in iron depletion), sFe (20 h in iron depletion and then supplemented with 10 μM FeSO_4_ for 1 h) and +Fe (20 h in 30 μM FeSO_4_) (**A**); grown at 37 °C in AMM −Fe and sFe for 15, 30 and 60 min (**B**); or grown at 37 °C in AMM −Fe and sFe for 10, 20 and 30 min with or without 50 μM MG132, dissolved in DMSO (**C**). Left panels: Representative Western blot analysis showing HapX protein levels. α-Tubulin was used as loading control. Right panels: Densitometric protein quantification. HapX protein levels were normalized to Tubulin and expressed relative to those in −Fe. Bars represent standard deviations from two independent biological experiments with two technical replicates each.

Iron starvation causes extensive transcriptional changes in fungi [11,12,13]. In *A*. *fumigatus*, like in other filamentous fungi, many of these changes are mediated by HapX [11,17] and have been studied in detail over the past two decades [23]. In order to study HapX at the protein level, we harvested *A*. *fumigatus* wild-type strain after growth in media reflecting iron starvation (−Fe), iron sufficiency (+Fe), and short-term adaptation from −Fe to +Fe (shift iron, sFe), i.e., 60 min after adding iron to iron-starved cultures. The quality of the samples, with regard to iron status, was tested by real time qRT-PCR of well-known iron-regulated genes: (i) induced in −Fe conditions, such as *hapX* itself, *mirB* (encoding a siderophore transporter), *sidF* and *sidH* (encoding enzymes required for extracellular siderophore biosynthesis); and (ii) induced in sFe and +Fe conditions, such as *sreA* (encoding a transcription factor required for iron uptake repression), *cycA* (encoding the cytochrome *c*), *cccA* (encoding a vacuolar iron transporter), and *hemA* (encoding a heme biosynthesis protein). Transcript levels of all the tested genes were as expected according to [11,16,17,20] (Figure S1A). For protein detection, we used a rabbit polyclonal antiserum raised against a peptide encompassing *A*. *fumigatus* HapX amino acid residues 161 to 491 [18]. Consistent with the expression of the gene, HapX is highly produced in −Fe but not in +Fe steady state cultures. Remarkably, we observed that most of the HapX signal was gone in the sFe condition sample, indicating that HapX is quickly degraded during the adaptation of *A*. *fumigatus* from iron-depletion to iron-sufficiency (Figure 2A).

To further investigate this regulation, we performed a sFe experiment analyzing samples collected in −Fe and 15, 30 and 60 min after the iron shift. Around 70% of HapX is degraded within the first 15 min demonstrating that the degradation process is fast (Figure 2B). Interestingly, HapX migrates as a band of higher molecular mass at this time point, suggesting that the protein is post-translationally modified after iron supplementation. The amount of HapX detected 30 and 60 min after the iron shift is even lower, about 15% of that found in −Fe; however, in these samples HapX mobility was not increased (Figure 2B). Next, we measured transcript levels of *hapX* and showed that most of its mRNA was lost 15 min after the iron shift slightly increasing at later time points (Figure S1B). For comparison, we tested protein stability of SidF and SidH, enzymes required for the biosynthesis of *A*. *fumigatus* extracellular siderophores fusarinine C (FsC) and triacetylfusarinine C (TAFC) [24,25], whose coding genes are transcriptionally co-regulated with *hapX* (Figures S1 and S2A). Detection of the two enzymes, using Venus-tagged strains denoted *^v^sidF* and *^v^sidH* (see material and methods) showed that, in contrast to HapX, proteins remained highly stable during the 1 h sFe experiment (Figure S2B).

Considering the fast degradation of HapX during sFe conditions, we tested whether the proteasome could be involved in this process. To address this question, we conducted a sFe experiment with and without the selective proteasome inhibitor MG132 [26] and showed that supplementation of this compound significantly reduced HapX degradation compared to the solvent control DMSO (Figure 2C).

Taken together, these results show that HapX is rapidly degraded in *A*. *fumigatus* during short-term adaptation from iron starvation to iron-replete conditions in a proteasome dependent manner.

### 2.2. HapX Turnover Is Very Fast during Iron Limitation

**Figure 3:**
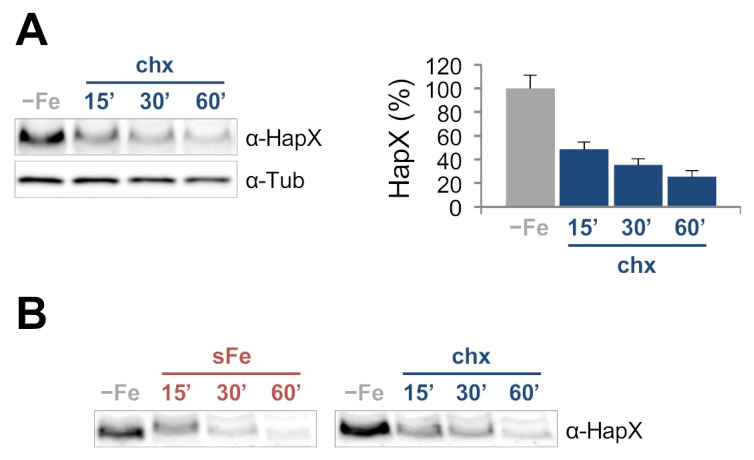
**HapX turnover is very fast in −Fe conditions.** (**A**,**B**). HapX quantification by Western blot analysis. Samples were obtained from the wild-type strain grown at 37 °C in AMM −Fe and then supplemented with 50 µg·mL^−1^ cycloheximide (chx) for 15, 30 and 60 min (**A**). Left panel: Representative Western blot analysis showing HapX protein levels. α-Tubulin was used as loading control. Right panel: Densitometric protein quantification. HapX protein levels were normalized to Tubulin and expressed relative to those in −Fe. Bars represent standard deviations from two independent biological experiments with two technical replicates each. (**B**). Comparison of sFe (Figure 1B) and chx (Figure 3A) samples separated in an 7.5% SDS-acrylamide gel for a better band size resolution.

To study HapX turnover rate under iron-limiting conditions, we analyzed its half-life by culturing the wild-type strain in −Fe conditions before adding the translation inhibitor cycloheximide (chx) [27], and taking samples 15, 30 and 60 min after the addition of the compound. Protein quantification showed that HapX levels decreased rapidly after chx treatment (Figure 3A); however, importantly, in contrast to the sFe experiment, the mobility of the protein remained unaltered during the whole experiment (Figure 3B). These results indicate that HapX turnover rate is extremely fast during iron limitation independently of the modifications seen under sFe conditions.

### 2.3. HapX Affinity Purification Studies Identified Protein Interactors and Post-Translational Modifications during the Adaptation from Iron-Deficiency to Iron-Replete Conditions

To study the HapX interactome and possible protein post-translational modifications taking place specifically during the adaptation from iron-depleted to iron-replete conditions, we cultured *A. fumigatus* wild-type strain and *^m^hapX*, a strain producing an N-terminal Myc-tagged HapX version (see material and methods), under sFe conditions and collected samples in −Fe and 10, 20 and 30 min after the iron shift. Subsequently, the corresponding crude cell extracts were subjected to Myc-Trap affinity purification [28]. Here, the wild-type was used as a negative control to discriminate unspecifically binding proteins. Importantly, *^m^hapX* is phenotypically indistinguishable from the wild-type strain (Figure S3A). Effective enrichment of ^M^HapX was validated by Western blot and nLC-MS/MS analysis (Figure S3B,C). Eluates from three independent biological Myc-Trap experiments were digested either with a Trypsin/LysC mixture or GluC proteases and subsequently analyzed by nLC-MS/MS.

#### 2.3.1. HapX Specifically Interacts with Fbx22 and SumO during sFe Conditions

**Figure 4:**
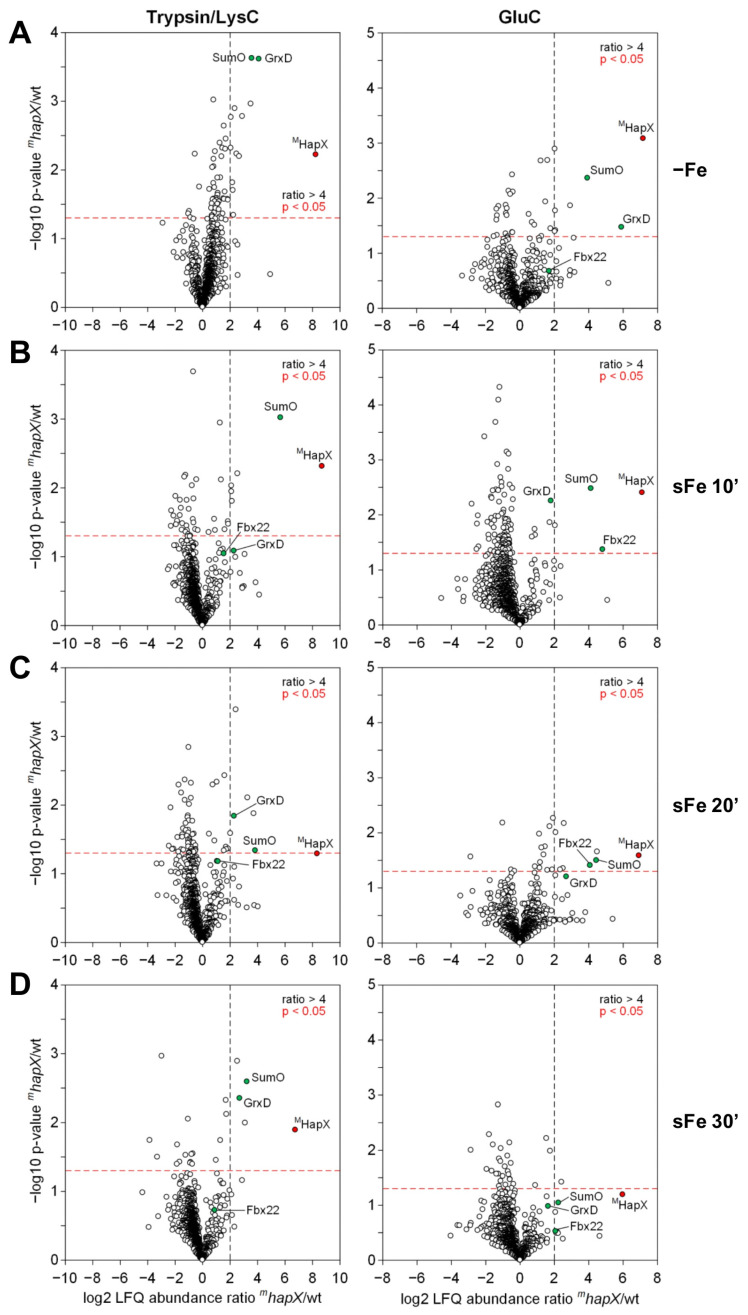
**HapX physically interacts with Fbx22 and SumO during the adaptation from iron-depleted to iron-replete conditions.** (**A**–**D**). Volcano-plot visualization of specifically enriched proteins interacting with ^M^HapX. Absolute label-free quantification (LFQ) abundances of proteins were determined by nLC-MS/MS analysis after Myc-Trap affinity purification from *A. fumigatus ^m^hapX* and plotted against LFQ abundances in wild-type eluates. Samples were obtained growing the strains at 37 °C in AMM −Fe (**A**) and sFe 10 (**B**), 20 (**C**) and 30 (**D**) min after the iron shift. Each dot represents the enrichment of a given protein vs. its reproducibility in three biological replicates. Specifically co-purified proteins are indicated by green dots. The bait, ^M^HapX, is marked as a red dot.

For visualization of specific HapX-interacting proteins, label-free quantification (LFQ) abundances of the most enriched proteins identified in *^m^hapX* Myc-Trap eluates were represented as volcano plots, displaying the enrichment of a given protein vs. its reproducibility in the three replicates, in comparison with the LFQ abundances in wild-type control eluates (Figure 4). As previously reported [18], and regardless of the digestion used after Myc-Trap affinity purification, we identified the monothiol glutaredoxin GrxD as one of the most highly enriched proteins under iron limitation (Figure 4A). GrxD, known to be essential for sensing iron starvation in *A. fumigatus* [18], was also detected, in lower abundance, in the three time points after the iron shift (Figure 4B–D). In addition to GrxD, we identified two previously uncharacterized interactors related to protein post-translational modification. The F-box protein Fbx22 (Afu6g13030) and the small ubiquitin-related modifier SumO (Afu1g10850) co-immunoprecipitated with ^M^HapX to different extents (Figure 4). F-box proteins, as part of the SCF complex (Skp, Cullin, F-box containing complex) [29,30], mediate the ubiquitination of proteins destined for the proteasomal degradation or other regulatory processes [31,32]. Fbx22 was co-purified during early time points after the iron shift and significantly co-enriched when the Myc-Trap eluates were digested with GluC (Figure 4B,C). On the other hand, SumO, a member of the small ubiquitin-like protein family of around 10 kDa able to modulate localization, activity, stability and interactions of the target proteins [33], was significantly enriched with independence of the digestion performed in −Fe conditions and, and with higher abundance, at early time points after the iron shift (Figure 4A–C).

Collectively, these results show that HapX interacts with partners involved in post-translational modification related to protein degradation and reinforces our previous finding of the proteasome implicated in HapX degradation.

##### Inactivation of Fbx22 and SumO Affects Iron Resistance, HapX Levels and Degradation and Heme Biosynthesis

**Figure 5:**
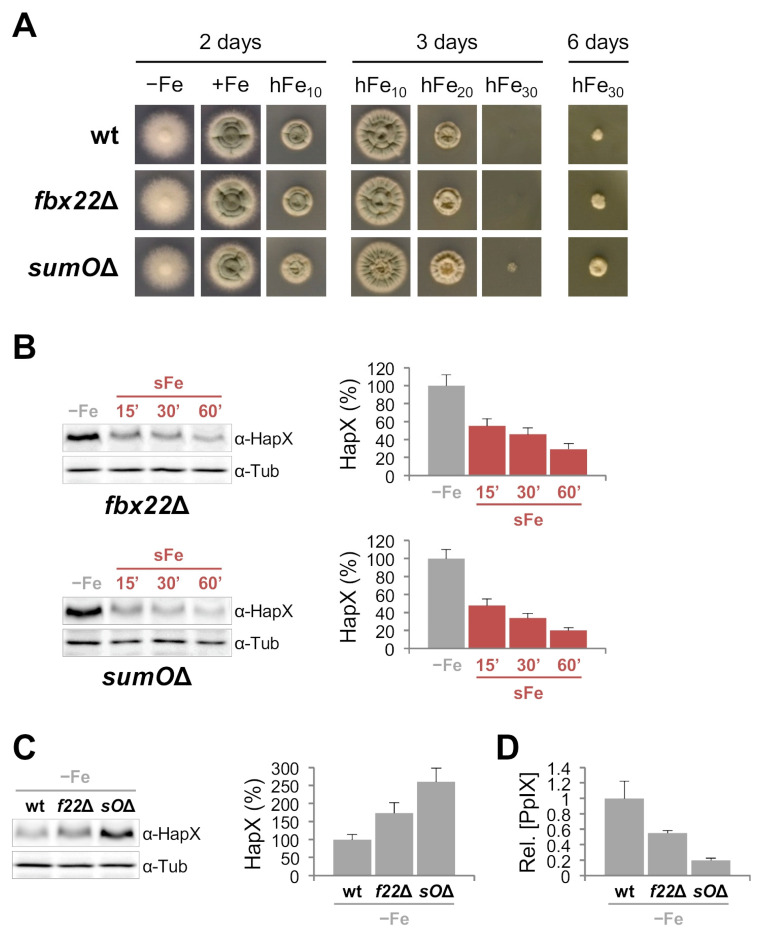
**SumO and Fbx22 are important for iron resistance, regulation of HapX protein levels and heme biosynthesis.** (**A**). Growth of the indicated strains on solid AMM −Fe (iron depletion), +Fe (30 µM FeSO_4_), and hFe (10, 20 or 30 mM FeSO_4_). Plates were incubated for the indicated time periods at 37 °C. Note that wild-type (wt) left panel is identical to that in Figure S3A. It is shown again here for clarity. (**B**,**C**). HapX quantification by Western blot analysis. Samples were obtained from the indicated strains grown under sFe conditions as in Figure 2B (**B**); or under −Fe conditions as in Figure 2A (**C**). Left panels: Representative Western blot analysis showing HapX protein levels. α-Tubulin was used as loading control. Right panels: Densitometric protein quantification. HapX protein levels were normalized to Tubulin and expressed relative to those in −Fe. (**D**). Quantification of PpIX in −Fe conditions. PpIX levels were normalized to the amount of total protein and expressed relative to those in the wild-type strain. Bars represent standard deviations from two independent biological experiments with two technical replicates each.

To study the role of Fbx22 and SumO in *A. fumigatus* HapX degradation, we generated *fbx22*Δ and *sumO*Δ strains replacing the entire coding sequences with the pyrithiamine resistance gene (see Material and Methods). To determine the impact of both mutations on growth during different levels of iron availability, we cultured *fbx22*Δ and *sumO*Δ, in comparison with the wild-type strain, in solid media under different iron conditions. *fbx22*Δ displayed no major phenotypical changes while, as shown for other *Aspergilli* [33,34,35], *sumO*Δ showed a small decrease in radial growth both under −Fe and +Fe conditions. Despite this, interestingly, *sumO*Δ and to a lesser degree *fbx22*Δ were slightly more resistant than the wild-type strain to highly toxic iron concentrations (hFe) (Figure 5A). Quantification of HapX levels during *A. fumigatus* adaptation from iron-depleted to iron-replete conditions showed that protein degradation was slightly less efficient in both mutants, with a difference somewhat higher in *fbx22*Δ (Figure 5B, see in comparison with Figure 2B). Importantly, the HapX band of higher molecular mass observed 15 min after the iron shift in the wild-type strain is still seen in both mutants (Figure 5B). Furthermore, we observed that −Fe HapX levels were increased about 1.75-fold and 3-fold in *fbx22*Δ and *sumO*Δ, respectively (Figure 5C). Since heme biosynthesis is de-repressed in *hapX*Δ under −Fe conditions [11,20], accumulation of its iron-free precursor protoporphyrin IX (PpIX) can be used as a readout of HapX activity under −Fe conditions. In line, PpIX biosynthesis, known to be increased in *hapX*Δ [11,20], decreased both in *fbx22*Δ and *sumO*Δ (Figure 5D). These results show that Fbx22 and SumO are important factors in the regulation of HapX protein levels, influencing both the production of important iron-related metabolites and iron resistance.

#### 2.3.2. HapX Is Ubiquitinated, Sumoylated and Phosphorylated during sFe Conditions

**Figure 6:**
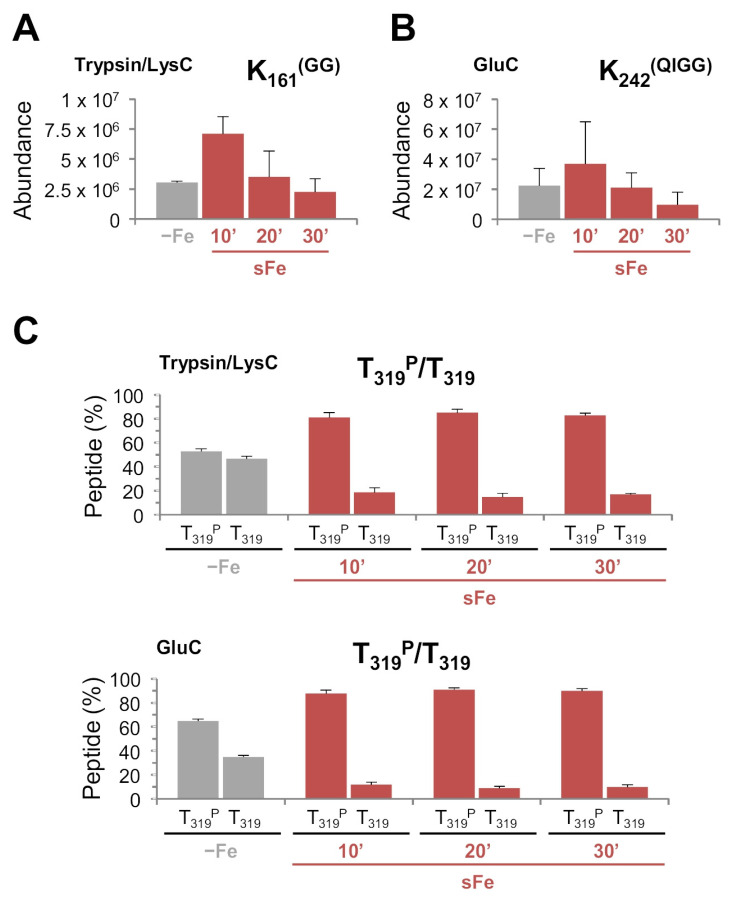
Ubiquitination of K161, sumoylation of K242, and phosphorylation of T319 are induced during a shift from iron-depleted to iron-replete conditions. (**A**–**C**) Label-free quantification of K161 ubiquitination (**A**), HapX K242 sumoylation (**B**), and T319 phosphorylation (**C**) determined by nLC-MS/MS analysis after Myc-Trap affinity purification from crude extracts of *^m^hapX* grown in AMM −Fe and then supplemented with 10 μM FeSO_4_ for 10, 20 and 30 min (sFe). Eluates were digested as indicated. Bars represent standard deviations from three independent biological experiments.

Considering the identified HapX interactome, we analyzed affinity-purified HapX peptides for ubiquitination and sumoylation signals. When Myc-Trap purified eluates were digested with the Trypsin/LysC mixture, a peptide containing K_161_^(GG)^, a ubiquitin remnant, was co-purified (Figure 6A). Vice versa, a peptide containing K_242_^(QIGG)^, a sumoylation remnant, was co-enriched when the eluates were digested with GluC (Figure 6B). Indeed, K242 was in silico identified as part of a negatively charged amino acid-dependent SUMOylation motif (NDSM) present in HapX (^241^IKPDPEEMEID^251^) [36]. Importantly, both modifications, like the interactions of HapX with Fbx22 and SumO, were co-enriched with the highest abundance 10 min after the iron shift (Figure 6A,B). In addition to these modifications, we identified several HapX peptides with phosphorylated residues (not shown); however, among them, only threonine (T) 319 was found phosphorylated in a relatively high proportion and differentially between −Fe vs. sFe conditions. Quantification of phosphorylated vs. non-phosphorylated peptides revealed that T319 phosphorylation was highly induced during sFe conditions (Figure 6C). These results suggest that the HapX post-translational modifications found during the adaptation of *A. fumigatus* from iron starvation to iron-sufficiency (shown in Figure 1) could be relevant in the regulation of the protein degradation process.

##### Point Mutations in HapX K161, K242 and T319 Affect Iron Resistance, HapX Levels and Degradation and Heme Biosynthesis

**Figure 7:**
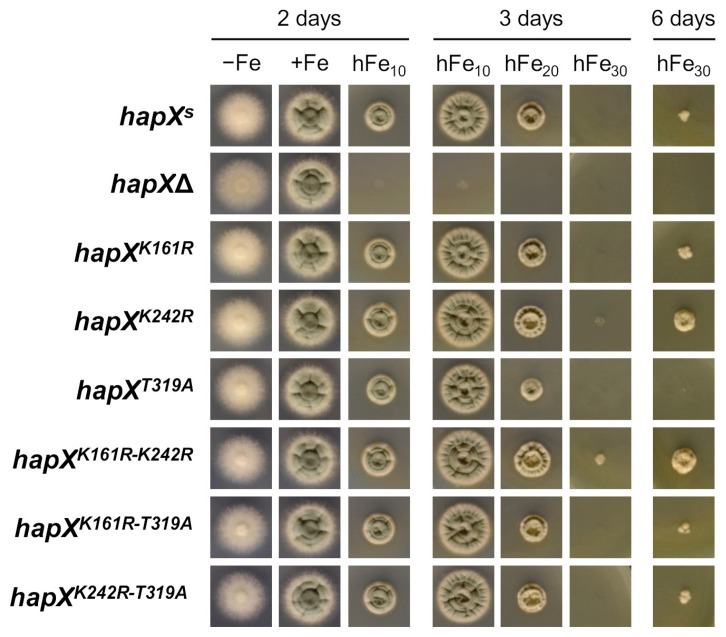
**Mutations in HapX K161, K242 and T319 affect iron resistance.** Growth of the indicated strains on solid AMM −Fe (iron depletion), +Fe (30 µM FeSO_4_), and hFe (10, 20 or 30 mM FeSO_4_). Plates were incubated for the indicated time periods at 37 °C.

To analyze the role of K161 ubiquitination, K242 sumoylation and T319 phosphorylation in HapX post-translational regulation, we generated the corresponding point-mutated strains taking advantage of the availability of the basic plasmid phapX^R^-hph, containing the *hapX* coding sequence C-terminally linked with the S-tag under the control of the native *hapX* promoter and terminator regions (see materials and methods) [17]. Therefore, our reference here is *hapX^s^*, a strain producing a C-terminal S-tagged HapX version showing no phenotypical differences with the wild-type strain (compare Figure 5A and Figure 7). K161 and K242 were substituted by arginine (R), thus preventing ubiquitination and sumoylation, respectively; while T319 was replaced by the non-phosphorylatable alanine (A). We denoted these strains *hapX^K161R^*, *hapX^K242R^* and *hapX^T319A^*. Moreover, we generated all possible double mutant combinations, including *hapX^K161R-K242R^*, *hapX^K161R-T319A^* and *hapX^K242R-T319A^*. Unlike the recipient strain *hapX*Δ [11], all the mutants showed no major growth defects on solid media under different iron conditions, indicating that these HapX versions are functional (Figure 7). Consistent with the phenotypes observed in *fbx22*Δ and *sumO*Δ (Figure 5A), *hapX^K161R^* and *hapX^K242R^* were slightly more resistant than the reference strain to hFe concentrations; however, in contrast, *hapX^T319A^* was more sensitive (Figure 7). Interestingly, we showed that the phenotype of the double mutants was, in all cases, determined by the combination of each of the single mutations. Note that hFe resistance is: (1) amplified in *hapX^K161R-K242R^* in comparison with any of the single mutants, (2) increased in *hapX^K161R-T319A^* and *hapX^K242R-T319A^* in comparison with *hapX^T319A^*, and (3) decreased in *hapX^K161R-T319A^* and *hapX^K242R-T319A^* in comparison with *hapX^K161R^* and *hapX^K242R^*, respectively (Figure 7).

**Figure 8:**
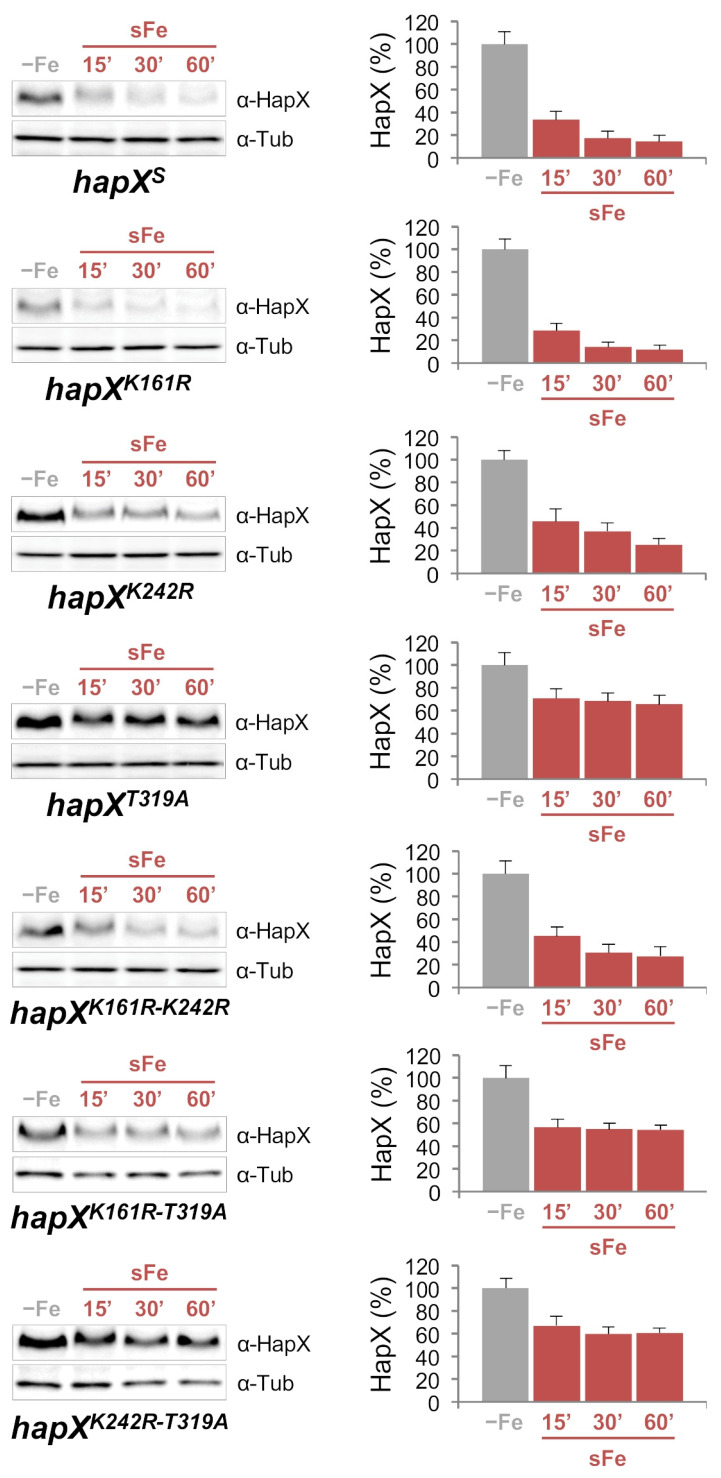
**Mutations in HapX K161, K242 and T319 affect HapX post-translational regulation.** HapX quantification by Western blot analysis. Samples were obtained from the indicated strains grown under sFe conditions as in Figure 2B. Left panels: Representative Western blot analysis showing HapX protein levels. α-Tubulin was used as loading control. Right panels: Densitometric protein quantification. HapX protein levels were normalized to Tubulin and expressed relative to those in −Fe. Bars represent standard deviations from two independent biological experiments with two technical replicates each.

**Figure 9:**
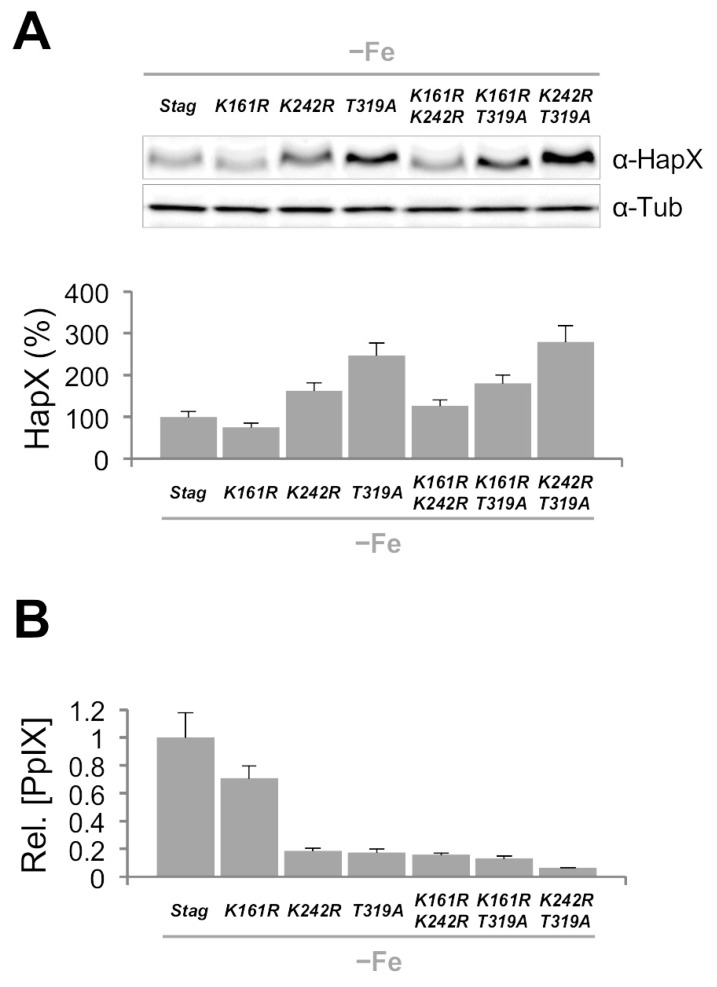
**K242 and T319 are important in the regulation of HapX protein levels.** (**A**). HapX quantification by Western blot analysis. Samples were obtained from the indicated strains grown under −Fe conditions as in Figure 2A. Upper panel: Representative Western blot analysis showing HapX protein levels. α-Tubulin was used as loading control. Lower panel: Densitometric protein quantification. HapX protein levels were normalized to Tubulin and expressed relative to those in −Fe. (**B**). Quantification of PpIX in −Fe conditions. PpIX levels were normalized to the amount of total protein and expressed relative to those in *hapX^s^.* Bars represent standard deviations from two independent biological experiments with two technical replicates each.

Quantification of HapX levels during the adaptation from iron starvation to iron sufficiency, fully comparable between *hapX^s^* and the wild-type strain (compare Figure 2B and Figure 8), revealed different patterns in all the tested mutants (Figure 8 and Figure 9A).

In *hapX^K161R^*, −Fe HapX levels were slightly decreased while sFe degradation was similar to that in *hapX^s^*; In *hapX^K242R^*, like in *sumO*Δ, −Fe HapX levels were increased and sFe degradation was slightly less efficient than in *hapX^s^*; and in *hapX^T319A^*, remarkably, −Fe HapX levels were highly increased whereas sFe degradation was tremendously affected in comparison with *hapX^s^*. Like in the plate assays, the double mutants displayed a HapX degradation pattern consistent with the combination of each of the single mutations (Figure 8). Significantly, the mobility difference observed in HapX 15 min after the iron shift was still detectable in all the mutants (Figure 8). It is important to highlight that in *hapX^T319A^*, HapX levels dropped slightly 15 min after the iron shift and remained stable from then on even when using a higher concentration of iron or a longer incubation time (Figure S4). To exclude the possibility that this phenomenon was due to an alteration of *hapX* transcriptional regulation, we measured transcript levels of *hapX*, and of the aforementioned HapX-regulated genes *mirB*, *sidF*, *sidH*, *sreA*, *cycA*, *cccA* and *hemA*, in *hapX^s^* and *hapX^T319A^* during sFe conditions. Expression of *hapX* was very similar in both strains and, with minor differences, they displayed a transcriptional response characteristic for the adaptation from iron-depleted to iron-replete conditions (Figures S5 and S6). Among the iron-repressed genes, it is remarkable that transcription of *sidF* and *sidH* was lower in *hapX^T319A^* vs. *hapX^s^* in −Fe (Figure S5) while, in general, iron-induced genes, such as *sreA*, *cycA*, *cccA* and *hemA* were more strongly activated in *hapX^T319A^* vs. *hapX^s^* under sFe conditions (Figure S6). A detailed comparative analysis of all the −Fe samples again showed that HapX levels were affected by the combination of each of the single mutations (Figure 9A). Thus, the production of PpIX decreased both in *hapX^K242R^* and *hapX^T319A^*, and in any double mutant with one of these two mutations (Figure 9B). Importantly, HapX migrates as a lower molecular mass band in *hapX^K161R^*, and in any double mutant with this mutation, indicating that K161 is post-translationally modified in −Fe conditions (Figure 9A). Taken together, these results show that ubiquitination of K161, sumoylation of K242 and phosphorylation of T319 are important post-translational modifications influencing hFe resistance, HapX protein levels and production of iron-related metabolites.

### 2.4. Overexpression of hapX Negatively Affects Growth and Production of Iron-Related Metabolites

**Figure 10:**
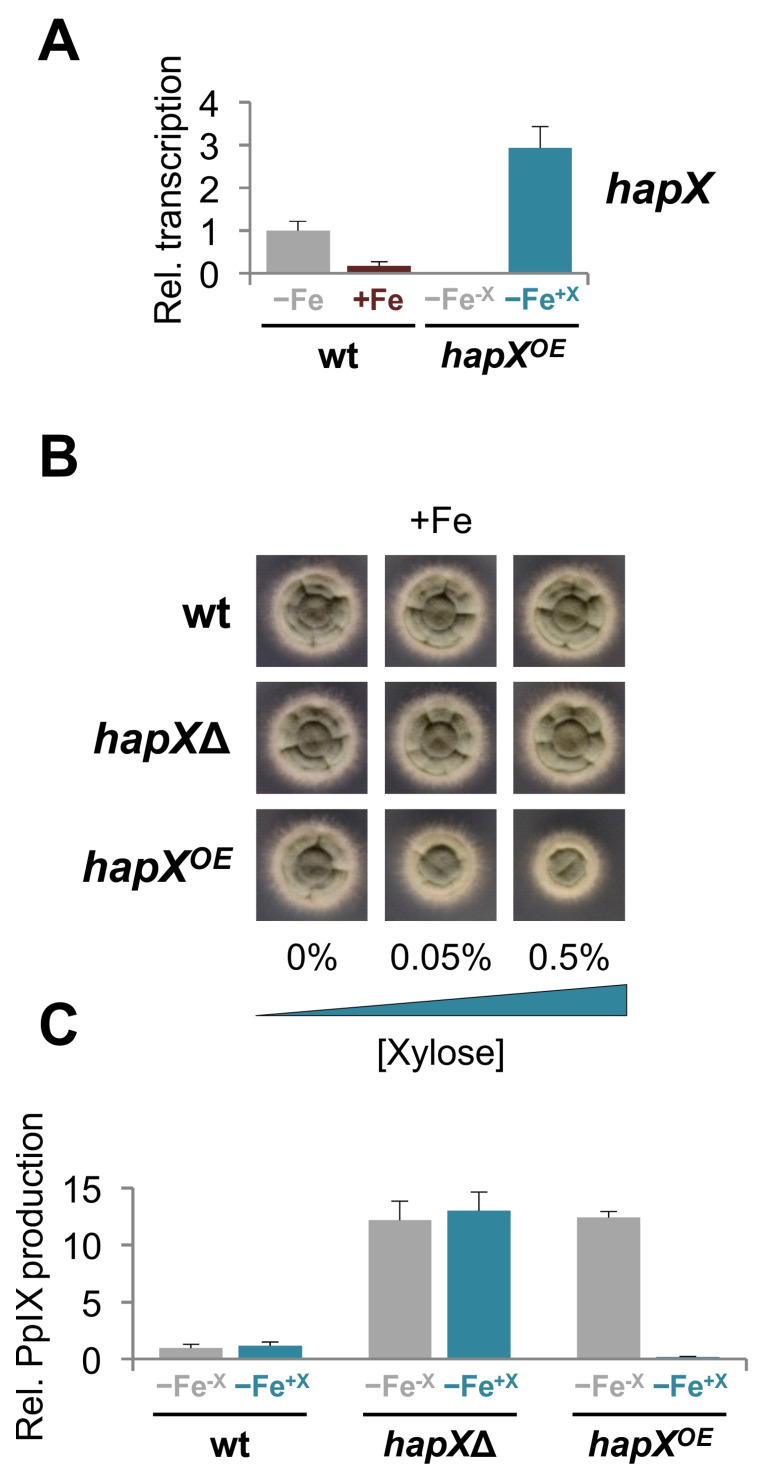
**Overexpression of *hapX* affects growth and PpIX production.** (**A**). Quantitative real-time reverse transcription RT-qPCR. Samples were obtained from the wild-type strain grown under −Fe and +Fe conditions as in Figure 2A, and from *hapX^OE^* grown in −Fe conditions as in Figure 2A (−Fe ^-X^) and then supplemented with 0.01% xylose for 15 min (−Fe ^+X^). Transcript levels of the indicated genes, normalized to *actA*, are expressed relative to those obtained in the wild-type strain in −Fe. Bars represent standard deviations from two independent biological experiments with two technical replicates each. (**B**). Growth of the indicated strains on solid AMM +Fe (30 µM FeSO_4_) with the indicated concentration of xylose (%). Plates were incubated for 2 d at 37 °C. (**C**). Quantification of PpIX in −Fe conditions with or without 0.05% xylose. PpIX levels of the indicated strains were normalized to the amount of total protein and expressed relative to those in the wild-type strain in the absence of xylose. Bars represent standard deviations from two independent biological experiments with two technical replicates each.

To analyze the impact of the concentration of HapX in *A*. *fumigatus*, we generated a strain in which *hapX* is under the control of the xylose inducible promoter *xylP^P^* [37], denoted here as strain *hapX^OE^* (see materials and methods). Quantitative real-time RT-PCR verified that *hapX* expression was controlled by the xylose concentration and not the iron status in *hapX^OE^* (Figure 10A). In accordance with the tight regulation of HapX observed during sFe conditions, overexpression of the gene (0.5% xylose) resulted in growth defects in +Fe cultures (Figure 10B). Slight *hapX* induction (0.05% xylose) gave rise to a wild-type like growth both under −Fe and hFe conditions, whereas large overexpression (0.5 and 1% xylose) was detrimental particularly under hFe conditions (Figure S7). This data again underlines the importance of strictly regulating HapX levels for iron homeostasis. Next, we measured accumulation of PpIX in *hapX^OE^* and, as expected, it was comparable to that in *hapX*Δ in the absence of xylose, and 10-fold reduced under overexpression conditions (Figure 10C). Collectively, these results show that increasing the HapX concentration provokes a severe impact on fungal growth and production of iron-related metabolites, highlighting the relevance of modulating HapX levels under different iron conditions.

## 3. Discussion

The CBC:HapX complex, discovered in the early 2000s [38] and molecularly characterized a few years later [20], is essential for the adaptation to different iron availability in filamentous fungi and yeasts [21,22,39,40,41,42]. Because HapX-mediated adaptation to iron deficiency is essential for virulence both in animal and plant pathogens [11,12,13,14,15], the study of this bZIP transcription factor has aroused a lot of interest. HapX is required for transcriptional activation of genes involved in iron acquisition as well as for repression of genes implicated in iron-dependent pathways [11,12,14,16]. Moreover, HapX was shown to be also essential for the adaptation to iron excess [17]. HapX CRR-A and CRR-B are crucial for this response while the C-terminal domain is responsible for the adaptation to iron starvation [17] (Figure 1). Over the past two decades, a lot of effort has been put into the investigation of HapX mode of action, transcriptional regulation and iron-sensing mechanism [18,19,21,23,39]; however, little is known about its regulation at the protein level. Here, we found that there is considerable post-translational regulation of HapX. To gain this insight, we studied *A. fumigatus* HapX regulation during short-term adaptation of from iron-depleted to iron-replete conditions, identifying new HapX interactors and post-translational modifications that will break a new scientific ground in our understanding of the regulation of this important transcription factor.

### 3.1. HapX Is Regulated at the Protein Level

HapX was rapidly degraded during sFe conditions (Figure 2B) while the extracellular siderophore biosynthetic enzymes SidF and SidH, whose coding genes are transcriptionally co-regulated with *hapX*, were not (Figure S2B). Importantly, we noticed that at early time points after the iron shift, HapX migrated as a protein of higher molecular mass (Figure 2B and Figure 3B), which indicates the existence of an iron-dependent post-translational modification mechanism. Furthermore, regardless of this iron-dependent regulation, we show that the half-live of HapX under −Fe conditions is also very short (Figure 3A), indicating that this transcription factor is subject to a complex regulation at the protein level. Protein levels within cells are determined by synthesis and degradation rates with half-lives varying greatly. It is known that many proteins with rapid turnover rates, required to allow their levels to change quickly in response to a stimulus, function as regulatory molecules, such as transcription factors [43]. Our results indicate that HapX is a clear example for such highly controlled regulatory proteins. This finding, together with the previously characterized tight transcriptional regulation [11,20], illustrates the necessity to regulate this transcription factor at multiple levels to strictly control iron homeostasis. MG132-mediated proteasome inhibition [26] prevented HapX degradation during sFe conditions (Figure 2C), which indicates that HapX is degraded, at least partially, in a proteasome dependent manner. It is important to note that the efficiency of the treatment with MG132 is transitory in *A. fumigatus* most likely due to the presence of multiple genes encoding ATP-binding cassette multidrug transporters in its genome. In yeast, it is known that inactivation of the drug efflux pump PDR5 increases sensitivity to MG132 [44].

### 3.2. HapX Interacts with Fbx22 and SumO and Is Prost-Translationally Modified Specifically under sFe Conditions

Our ^M^HapX Myc-Trap affinity purification assay followed by nLC-MS/MS-based quantification of the most enriched proteins during sFe conditions identified, apart from the previously reported monothiol glutaredoxin GrxD [18], two uncharacterized HapX interactors, Fbx22 and SumO, mainly in extracts from early time points after iron supplementation (Figure 4). In line with these results two HapX peptides, one containing K_161_^(GG)^, a ubiquitin remnant, and another containing K_242_^(QIGG)^, a sumoylation remnant, were co-enriched when eluates were digested with the Trypsin/LysC mixture and GluC, respectively (Figure 6A,B). This agrees with the unsuitability of using Trypsin/LysC for MS/MS detection of sumoylated peptides (Figure S8) and GluC for detection of ubiquitinated peptides due to the large size of the remaining attached peptides [45]. Notably, our amino acid sequence alignment of the C-terminal region of *Aspergillus* species orthologs (Figure S8) revealed that except *A. nidulans* and *A. terreus* SumO, all other mature SumO proteins lack a short C-terminal extension of amino acids that has to be removed by SUMO peptidases prior to covalent substrate binding via the C-terminal diglycine residue motif. This fact has already been noticed for *A. flavus* SumO [46].

The F-box protein Fbx22, recently found to be involved in carbon catabolite repression responses in the model fungus *Neurospora crassa* [47], is a putative ortholog of *Saccharomyces cerevisiae* Cdc4p, required for G1/S and G2/M phase transitions [48]. Since iron is known to be a major regulator of the cell cycle with both situations, iron limitation and iron excess, related to cell cycle arrest [49], and considering the effect of MG132 on HapX degradation, the HapX-Fbx22 interaction perfectly fits with our experimental data: Fbx22 would interact with HapX, preferentially in the short-term adaptation from iron starvation to iron sufficiency, to promote ubiquitination of the latter and its subsequent degradation, thereby preventing excessive iron accumulation in the cell. Indeed, HapX degradation is delayed in *fbx22*Δ vs. the wild-type strain under sFe conditions (Figure 5B), but it is still possible. This, along with the observation that HapX still maintains iron-dependent post-translational modification (note the higher molecular mass HapX band immediately after iron addition), indicates that additional mechanisms/players are regulating this process (see below). Furthermore, HapX −Fe content was higher in *fbx22*Δ vs. the wild-type strain, consequently affecting PpIX production (Figure 5C,D). This finding suggests that Fbx22 plays a role in HapX turnover under −Fe conditions. HapX degradation during sFe conditions was very similar in *hapX^K161R^* vs. its reference strain (*hapX^S^*) (Figure 8). It is possible that, instead of signaling HapX for degradation, ubiquitination of K161 plays a role in regulation of the protein structure, protein-protein interaction and/or DNA/chromatin interaction. Although we did not detect any additional ubiquitinated lysines in HapX, such assumption is plausible and perhaps the reason why we did not observe changes in the HapX sFe degradation pattern in *hapX^K161R^*. In line, it is known that several F-box proteins, such as Cdc4, can ubiquitinate their target proteins on numerous lysines [50] and that interfering with one in particular may have no major impact. Although K161 is preferentially ubiquitinated at early time points after iron supplementation (Figure 6A), this residue is already post-translationally modified under −Fe conditions. Note that HapX^K161R^ migrates as a protein of lower molecular mass in comparison with HapX^S^ (Figure 9A). The fact that the size of HapX is similar in *fbx22*Δ and the wild-type strain under −Fe conditions (Figure 5C) indicates that Fbx22 is not required for −Fe post-translational modification of K161. Whether K161 is not ubiquitinated via Fbx22, or whether Fbx22 can be replaced by another Fbx protein in *fbx22*Δ needs to be clarified. Importantly, inactivation of CDC4/Fbx22 is lethal in yeast [51], which might indicate that Fbx22 functions can be fulfilled by alternative Fbx proteins in filamentous fungi. On the other hand, we cannot rule out the possibility that HapX is ubiquitinated at other lysine residues via Fbx22. Interestingly, *fbx22*Δ and *hapX^K161R^* are both slightly more resistant to hFe conditions than their reference strains (Figure 5A and Figure 7) suggesting that the role of Fbx22 and the ubiquitination of HapX K161 are functionally connected.

Transcription factors are among the most frequently detected targets of sumoylation [52,53]. Unlike in *S. cerevisiae*, where inactivation of SUMO is lethal [34], *sumO* can be deleted in *Aspergilli* and *Schizosaccharomyces pombe* giving rise to drastic phenotypical changes [33,34,54]. The relevance of HapX sumoylation is underlined by the fact that both *sumO*Δ and *hapX^K242R^* were more resistant to hFe conditions (Figure 5A and Figure 7). Furthermore, HapX degradation was slightly less efficient during sFe conditions (Figure 5B and Figure 8), while −Fe HapX levels were increased, resulting in reduced PpIX accumulation (Figure 5C,D and Figure 9), in both strains. Consistent with the finding that sumoylation often decreases transcription factor-chromatin association to regulate gene expression [52], our results indicate that sumoylation reduces HapX activity. It is important to note that HapX mobility during −Fe conditions was comparable between *sumO*Δ and the wild-type strain and between *hapX^K242R^* and *hapX^S^* (Figure 5C and Figure 9A). This indicates that, unlike K161 ubiquitination, sumoylation of K242 occurs almost exclusively under sFe conditions. Interestingly, *hapX^K161R-K242R^*, a non-K161-ubiquitinable and non-K242-sumoylatable strain, displayed an intermediate phenotype between those observed for each of the single mutations regarding HapX −Fe content and mobility (Figure 9A) and resistance to hFe concentrations (Figure 7). Therefore, both signals independently regulate HapX function in *A. fumigatus*.

Besides ubiquitination and sumoylation, we found that HapX T319 is phosphorylated preferentially under sFe conditions (Figure 6C). Replacement of HapX T319 with alanine dramatically increased HapX −Fe content (Figure 8 and Figure 9A), thereby causing decreased PpIX accumulation (Figure 9B), even though *hapX* transcript levels during iron starvation were similar in *hapX^T319A^* vs. its reference strain (Figure S5). Furthermore, HapX^T319A^ degradation during sFe conditions was extraordinarily diminished (Figure 8 and Figure S4). These results indicate that phosphorylation of T319 is essential for the maintenance of the HapX turnover rate and for HapX degradation under sFe conditions. Moreover, tolerance to hFe concentration was reduced in *hapX^T319A^* (Figure 7) highlighting the relevance of regulating the level of HapX in response to different iron conditions. In agreement with this observation, overexpression of *hapX* negatively affected *A. fumigatus* growth under +Fe and hFe conditions (Figure 10B and Figure S7). *hapX^K161R-T319A^* and *hapX^K242R-T319A^* exhibited a mixture of each of the individual mutations concerning HapX −Fe levels and mobility (Figure 9A), HapX degradation during sFe conditions (Figure 8), and resistance to hFe concentrations (Figure 7). This suggests that regulation of HapX via phosphorylation of T319 acts independently of K161 ubiquitination and K242 sumoylation in *A. fumigatus*. Whether there is crosstalk between the detected reversible post-translational modifications or between these and others that we may have not detected to regulate HapX function and stability remains to be elucidated. For example, it is known that phosphorylation can promote ubiquitination that can lead to proteasomal degradation [55]. The effect of MG132 treatment and T319A substitution on HapX degradation during sFe conditions points towards this direction. Considering all our results, we postulate that K161 ubiquitination and K242 sumoylation mainly regulate HapX activity while T319 phosphorylation regulates HapX stability.

One important question is how it is mechanistically possible that these residues, K161, K242 and T319, are modified within such a short time period. Because HapX is itself an iron sensor, most likely via [2Fe-2S] cluster binding [18,19], it is possible that the modifying apparatus recognizes the conformational change that HapX undergoes upon binding of [2Fe-2S] clusters. This conformational change could make the recognition motifs harboring K161, K242 and T319 more accessible for the respective modifications. In any case, this study clearly demonstrates that HapX is subject to complex post-translational modifications in *A. fumigatus*.

## 4. Materials and Methods

### 4.1. Fungal Isolates and Culture Conditions

*Aspergillus fumigatus* AfS77 (*akuA*Δ strain derived from ATCC46645 lacking non-homologous recombination) was used in all experiments [56] cultured at 37 °C for the indicated time periods. Fungal strains were stored as conidial suspensions at −80 °C with 30% glycerol. All strains used in this study are listed in Table S1. For liquid cultures, 10^6^ conidia mL^−1^ were cultured at 200 rpm in *Aspergillus* minimal medium (AMM) [57] containing 1% glucose (*w*/*v*) and 20 mM glutamine as carbon and nitrogen sources, respectively. For solid cultures, 2 × 10^4^ conidia were spotted onto AMM agar or agarose (to avoid iron contaminations) plates. Iron (FeSO_4_) concentrations used in each experiment are indicated in the figures. To inhibit the proteasome, MG132 (Merck KGaA, Darmstadt, Germany) was used at a final concentration of 50 µM. To block protein biosynthesis, 50 µg·mL^−1^ of the translation inhibitor cycloheximide (chx) (Merck KGaA, Darmstadt, Germany) we used. In *hapX^OE^*, *hapX* transcription was induced with the indicated concentrations of xylose (*w*/*v*).

### 4.2. Generation of Mutant Strains

For *venus*-tagging of *sidF* and *sidH* (*^v^sidF* and *^v^sidH*), cassettes, including each coding sequence N-terminally linked with the *venus*-tag under the control of their native promoter and terminator region as well as the hygromycin resistance marker, were assembled by fusion PCR [58] and protoplasts of *A. fumigatus* AfS77 were transformed to hygromycin resistance.

For *myc*-tagging of *hapX* (*^m^hapX*), the cassette, including the *hapX* coding sequence N-terminally linked with *myc*-tag under the control of its native promoter and the *nos*-terminator (*nosT*) region as well as the hygromycin resistance marker, was obtained by Gibson assembly and was performed using the NEBuilder^®^ HiFi DNA Assembly Master Mix (New England Biolabs, Frankfurt, Germany) [59]. The cassette was then used to transform a *hapX*Δ strain in which the pyrithiamine resistance gene had been used to replace the *hapX* coding sequence [17].

For the generation of *A. fumigatus fbx22* (Afu6g13030) and *sumO* (Afu1g10850) deletion mutant strains, targeted replacement cassettes were constructed amplifying DNA fragments flanking both coding sequences and fusing them with the pyrithiamine resistance marker by Gibson assembly [59]. Subsequently, protoplasts of *A. fumigatus* AfS77 were transformed to pyrithiamine resistance.

For the generation of *hapX^K161R^*, *hapX^K242R^*, *hapX^T319A^*, *hapX^K161R-K242R^*, *hapX^K161R-T319A^* and *hapX^K242R-T319A^* mutant strains, we used the basic plasmid phapX^R^-hph [17]. In order to substitute specific amino acids by site-directed mutagenesis, the QuickChange Site-Directed Mutagenesis Kit (Aligent, Santa Clara, CA, USA) was used. For the introduction of each mutation, phapX^R^-hph was amplified with complementary primers including the desired change. Resulting plasmids were confirmed by sequencing. Plasmids were then linearized and used to transform a *hapX*Δ strain in which the pyrithiamine resistance gene had been used to replace the *hapX* coding sequence [17].

For conditional expression of *hapX* (*hapX^OE^*), we generated a plasmid containing the *hapX* coding sequence C-terminally linked with an S-tag [17] under the control of *xylP^P^*, a xylan/xylose-inducible promoter derived from the xylanase *xylP* of *Penicillium chrysogenum* [37], as well as the hygromycin resistance marker. The plasmid was then linearized and used to transform a *hapX*Δ strain in which the pyrithiamine resistance gene had been used to replace the *hapX* coding sequence [17].

In all cases, transformants showing homologous insertions were genotyped by PCR and Southern blot analysis (not shown).

### 4.3. Nucleic Acid Manipulations, Quantitative Real-Time RT-PCR and PpIX Analysis

Total RNA and gDNA were extracted from *A*. *fumigatus* mycelia following previously reported protocols [60]. Quality and quantity of extracted nucleic acids were determined by running aliquots in ethidium bromide-stained agarose gels and by spectrophotometric analysis in a NanoDrop ND-1000 spectrophotometer (NanoDrop Technologies, Wilmington, DE, USA), respectively. Quantitative RT-PCR was performed as described previously [12,61] using FastStart Essential DNA Green Master (Roche Diagnostics SL, Barcelona, Spain) in a CFX Connect Real-Time System (Bio-Rad, Madrid, Spain). Gene specific primers were designed to flank an intron, if possible. Transcript levels were calculated by comparative Δ*Ct* and normalized to *actA*. PpIX production analysis was carried out as described previously [20].

### 4.4. Western Blotting

Proteins were extracted using a reported procedure [62] involving solubilization from lyophilized mycelial biomass with NaOH, followed by precipitation with trichloroacetic acid (TCA). Aliquots were resolved in 10-12% SDS-polyacrylamide gels (Carl Roth GmbH + Co. KG, Karlsruhe, Germany) and transferred to nitrocellulose membranes with a Trans-Blot Turbo Transfer System (Bio-Rad, Madrid, Spain) for blotting. Western blots were reacted with anti-HapX antisera (1:20,000; Davids Biotechnologie, Regensburg, Germany) or with polyclonal anti-GFP (1:10,000; A11122 ThermoFisher Scientific Inc., Waltham, MA, USA) as primary antibodies and with monoclonal anti-rabbit IgG peroxidase (1:10,000; A1949 Merck KGaA, Darmstadt, Germany) as secondary antibody. Tubulin, used as loading control, was detected with monoclonal anti-α-tubulin (1:10,000; T6119 Merck KGaA, Darmstadt, Germany) as primary antibody and with polyclonal anti-mouse IgG peroxidase (1:10,000; A4416 Merck KGaA, Darmstadt, Germany) as secondary antibody. Proteins were detected with ECL (Thermo Fisher Scientific, Waltham, MA, USA) and densitometric quantifications were performed with ImageJ 1.48v (National Institutes of Health, Bethesda, MD, USA).

### 4.5. Myc-Trap Immunoprecipitation of ^M^HapX Fusion Protein

*A. fumigatus* mycelia were harvested in Stop buffer [63] at 4 °C after growth for 22 h and freeze-dried. Protein extraction was performed according to a modified procedure from [63] using HK buffer containing 10 µM *N*-Ethylmaleimide (NEM) (Merck KGaA, Darmstadt, Germany) for total protein extraction. All steps were carried out at 4 °C in the cold room. In brief, 100 mg of freeze-dried mycelium powder were ground in a MixerMill MM 400 (Retsch GmbH, Haan, Germany) for 1 min at 30 Hz and then digested again in 1 mL HK buffer for 1 min at 30 Hz. Cell extracts were centrifuged twice at 20,187× *g* for 15 min and 500 µL of the supernatant was incubated with Myc-Trap^®^ agarose beads (ChromoTek GmbH, Planegg-Martinsried, Germany) for 1 h. The beads were washed twice in HK buffer without NP40, twice in wash buffer (25 mM Tris/HCl pH 7.5, 500 mM NaCl, 5 mM EDTA and 15 mM EGTA) and once in ultrapure water. Proteins were eluted in 10% (*v*/*v*) acetonitrile and 5% (*v*/*v*) acetic acid and used for nLC-MS/MS measurement and Western blot detection.

### 4.6. In-Solution Digest of Myc-Trap Eluates

Dried Myc-Trap eluates were solubilized in 50 µL 50 mM NH_4_HCO_3_ in 50:50 (*v*/*v*) trifluoroethanol (TFE)/water. After heat denaturation (90 °C, 10 min) the proteins were reduced for 1 h at 55 °C by addition of TCEP (Tris(2-carboxyethyl)phosphine) at a final concentration of 8 mM. Further carbamidomethylation was performed for 45 min at 32 °C in 15 mM chloroacetamide. Subsequently the samples were evaporated in a vacuum concentrator (Eppendorf, Wesseling, Germany) to a residual volume of approximately 5 µL. Finally, the volume was set to 30 µL with 50 mM NH4HCO3 and proteins were digested overnight (18 h, 37 °C) with a Trypsin/LysC mixture (Promega, Walldorf, Germany) as well as GluC endoproteinase (Promega, Walldorf, Germany) at a protein to protease ratio of 25:1. The digestion was stopped by adding 10% (*v*/*v*) formic acid and peptides were dried in a vacuum concentrator (Eppendorf, Wesseling, Germany), re-solubilized in 20 µL of 0.05% TFA in H_2_O/acetonitrile 98/2 (*v*/*v*) and filtered through a 10 kDa spin filter. The filtrate was transferred to HPLC vials and injected into the LC-MS/MS instrument. Each sample was measured in triplicate (3 analytical replicates).

### 4.7. nLC-MS/MS Analysis

nLC-MS/MS analysis was carried out on an Ultimate 3000 nano (n) RSLC system coupled to a QExactive Plus mass spectrometer (both Thermo Fisher Scientific, Waltham, MA, USA). Peptides were trapped for 5 min on an Acclaim Pep Map 100 column (2 cm × 75 µm, 3 µm) at 5 µL/min followed by gradient elution separation on an Acclaim Pep Map RSLC column (50 cm × 75 µm, 2 µm). Eluent A (0.1% (*v*/*v*) formic acid in water) was mixed with eluent B (0.1% (*v*/*v*) formic acid in 90/10 acetonitrile/water) as follows: 0 min at 4% B, 6 min at 6% B, 14 min at 10% B, 20 min at 14% B, 35 min at 20% B, 42 min at 26% B, 46 min at 32% B, 52 min at 42% B, 55 min at 50% B, 58min at 65% B, 60–64.9 min at 96% B, 65–90 min at 4% B. Positively charged ions were generated at 2.2 kV using a stainless steel emitter and a Nanospray Flex Ion Source (Thermo Fisher Scientific, Waltham, MA, USA). The QExactive Plus was operated in Full MS/data-dependent MS2 (Top10) mode. Precursor ions were monitored at *m*/*z* 300–1500 at a resolution of 70,000 FWHM (full width at half maximum) using a maximum injection time (ITmax) of 120 ms and an AGC (automatic gain control) target of 1 × 10^6^. Precursor ions with a charge state of z = 2–5 were filtered at an isolation width of *m*/*z* 1.6 amu for HCD fragmentation at 30% normalized collision energy (NCE). MS2 ions were scanned at 17,500 FWHM (ITmax = 120 ms, AGC = 2 × 10^5^). Dynamic exclusion of precursor ions was set to 20 s. The LC-MS/MS instrument was controlled by QExactive Plus Tune 2.9 and Xcalibur 3.0 with DCMS Link.

### 4.8. Protein Database Search

Tandem mass spectra were searched against the *Aspergillus* Genome Database (AspGD) of *Aspergillus fumigatus* Af293 (http://www.aspergillusgenome.org/download/sequence/A_fumigatus_Af293/current/A_fumigatus_Af293_current_orf_trans_all.fasta.gz (accessed on 4 February 2019)) and the protein sequence of Dre2 (AFUB_008090; the Dre2 ortholog is not present in the Af293 gene annotation) as well as further modified protein sequences (e.g., Myc-tag) using Proteome Discoverer (PD) 2.2 (ThermoFisher Scientific Inc., Waltham, MA, USA) and the algorithms of Sequest HT (version of PD2.2) and MS Amanda 2.0. Two missed cleavages were allowed for the proteolytic digestions. The precursor mass tolerance was set to 10 ppm and the fragment mass tolerance was set to 0.02 Da. Modifications were defined as dynamic oxidation of Met, acetylation of Ser, phosphorylation of Ser, Thr, and Tyr, ubiquitination (GG) and sumoylation (QIGG) of Lys as well as static Cys carbamidomethylation. At least two peptides per protein and a strict false discovery rate (FDR) < 1% were required for positive protein hits. The Percolator node of PD2.2 and a reverse decoy database was used for q-value validation of spectral matches. Only rank 1 proteins and peptides of the top scored proteins were counted. The Minora algorithm of PD2.2 was applied for relative label-free quantification. Myc-Trap eluates from wt *A. fumigatus* mycelial extracts were used for quantification of nonspecifically co-purified proteins. Potential HapX-interacting proteins were determined using the two-sided *t*-test and visualized by volcano plots.

### 4.9. Western Blot Detection of Proteins after Myc-Trap

Proteins were separated by SDS-PAGE using NuPAGE™ 4-12% (*w*/*v*) Bis-Tris gradient gels (ThermoFisher Scientific Inc., Waltham, MA, USA). For Western detection, proteins were transferred onto a PVDF membrane using the iBlot™ 2 dry blotting system (ThermoFisher Scientific Inc., Waltham, MA, USA). The membrane was blocked in 3% (*w*/*v*) bovine serum albumin (BSA) dissolved in 1x PBST (137 mM NaCl, 2.7 mM KCl, 10 mM Na_2_HPO_4_, 2 mM KH_2_PO_4_, 0.05% (*v*/*v*) Tween 20). Western blots were reacted with anti-HapX antisera (1:10,000; Davids Biotechnologie, Regensburg, Germany) or with polyclonal anti-c-Myc (1:1000; ab9106 Abcam plc, Cambridge, UK) as primary antibodies and with monoclonal anti-rabbit IgG peroxidase (1:10,000; A1949 Merck KGaA, Darmstadt, Germany) as secondary antibody. The membrane was developed using the 1-Step™ Ultra TMB-Blotting chromogenic substrate (ThermoFisher Scientific Inc., Waltham, MA, USA).

## Data Availability

The mass spectrometry proteomics data have been deposited to the ProteomeXchange Consortium via the PRIDE [64] partner repository with the dataset identifier PXD026889.

## Supplementary Materials

Figure S1. Transcription of iron-regulated genes. Figure S2. SidF and SidH are stable during sFe conditions. Figure S3. Effective enrichment of ^M^HapX validated by Western blot and nLC-MS/MS analysis. Figure S4. Quantification of HapX in *hapX^s^* and *hapX^T319A^* under different sFe conditions. Figure S5. Transcription of iron-repressed genes. Figure S6. Transcription of iron-induced genes. Figure S7. Effect of HapX overexpression on −Fe and hFe colony growth. Figure S8. Amino acid sequence alignment of the C-terminal region of SumO orthologs from various *Aspergillus* species. Table S1. Strains used in this study.

## Abbreviations

RIA	Reductive iron assimilation
CBC	CCAAT-binding complex
CRR	Cysteine rich region
−Fe	Iron-deplete
+Fe	Iron-replete
sFe	Shift from iron-depleted to iron-replete
FsC	Fusarinine C
TAFC	Triacetylfusarinine C
chx	Cycloheximide
LFQ	Label-free quantification abundances
SCF	Skp, Cullin, F-box
hFe	high iron
PpIX	Protoporphyrin IX
NDSM	Negatively charged amino acid-dependent SUMOylation motif
AMM	*Aspergillus* minimal medium
TCA	Trichloroacetic acid
