# Supplementary material for: The bZIP Transcription Factor HapX Is Post-Translationally Regulated to Control Iron Homeostasis in Aspergillus fumigatus
Source: Int J Mol Sci. 2021 Jul 20;22(14):7739. doi: 10.3390/ijms22147739 (PMC8307855; doi:10.3390/ijms22147739)
Supplement: Supplementary file 1 [file ijms-22-07739-s001.zip › ijms-1308239-supplementary.pdf]

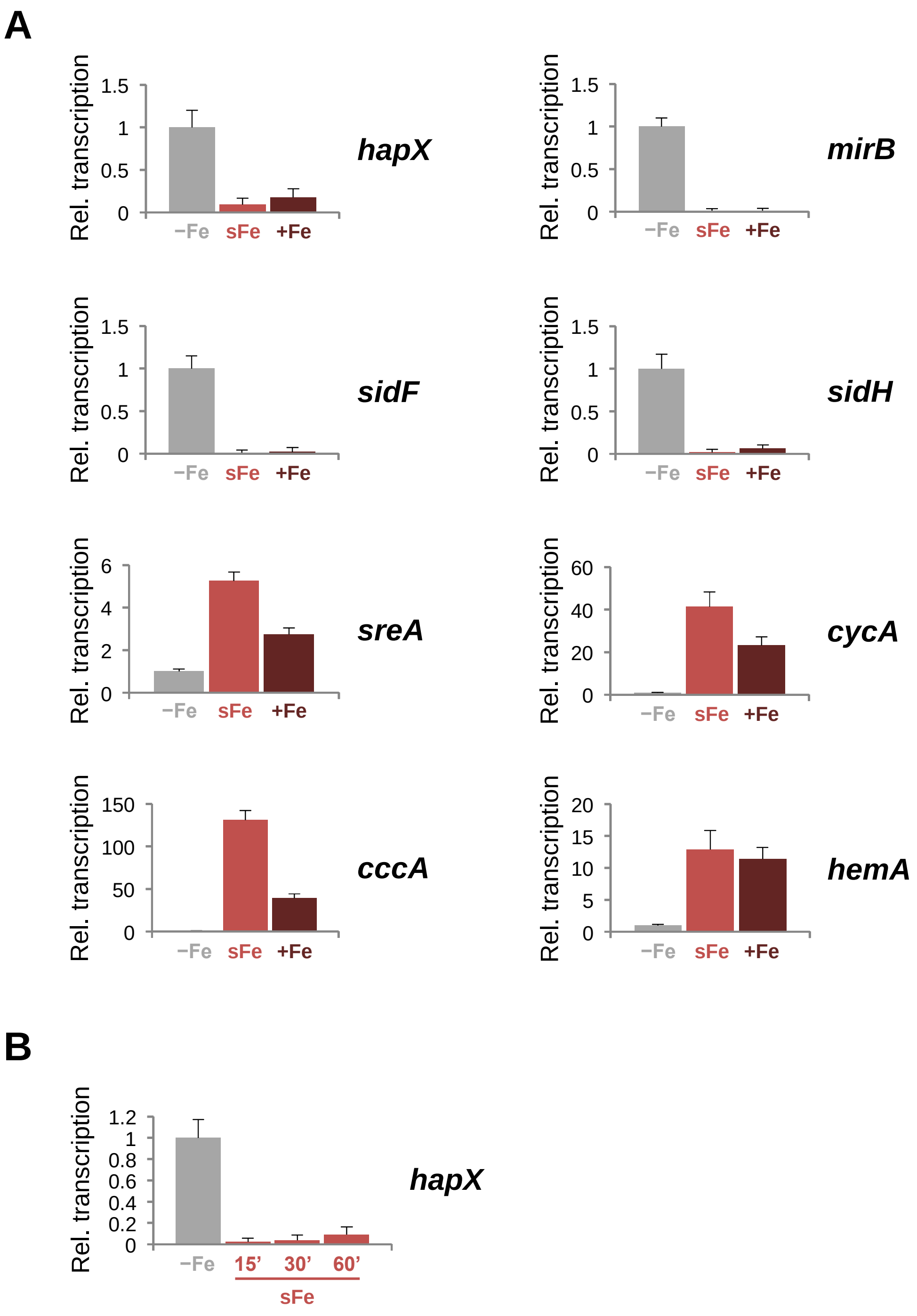

Figure S1. Transcription of iron-regulated genes.

Quantitative real-time reverse transcription RT-qPCR. Samples were obtained from the wild-type strain grown at 37ºC in liquid Aspergillus Minimal Medium (AMM) −Fe (20 h in iron depletion), sFe (20 h in iron depletion and then supplemented with 10 μM $FeSO_4$ for 1 h) and +Fe (20 h in 30 μM $FeSO_4$) **(A)**; or grown at 37ºC in AMM −Fe and sFe for 15, 30 and 60 min **(B)**. Transcript levels of the indicated genes, normalized to *actA*, are expressed relative to those obtained in −Fe. Bars represent standard deviations from two independent biological experiments with two technical replicates each.

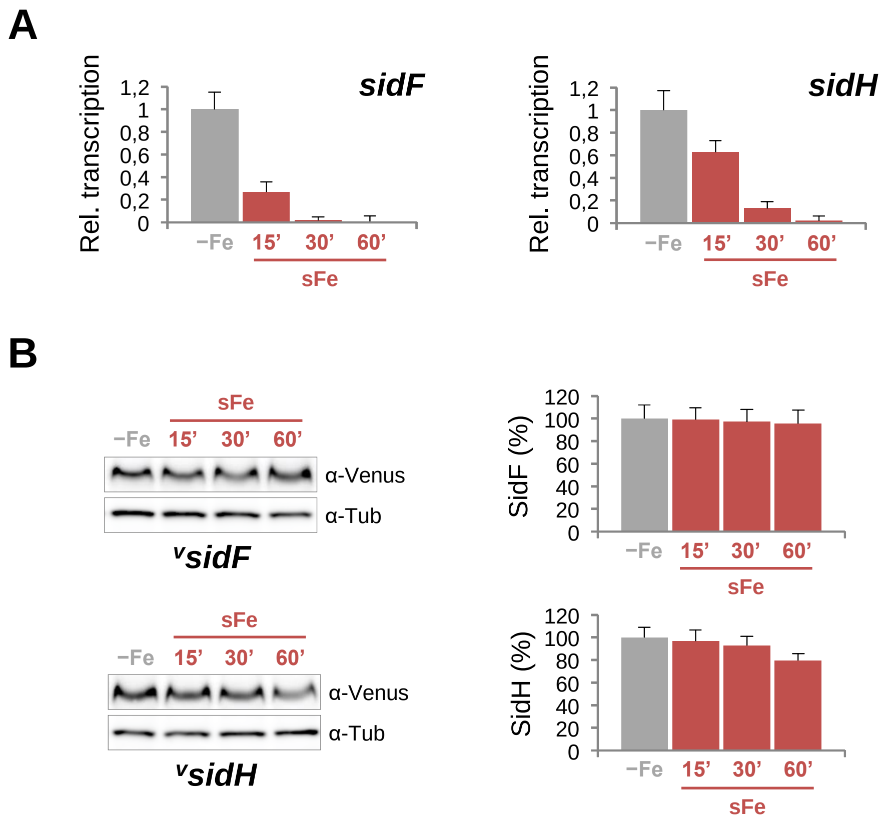

Figure S2. SidF and SidH are stable during sFe conditions.

A. Quantitative real-time reverse transcription RT-qPCR. Samples were obtained from the wild-type strain grown at 37ºC in iron-depleted liquid AMM for 20 h (−Fe) and then supplemented with 10 μM $FeSO_4$ for 15, 30 and 60 min (sFe). Transcript levels of the indicated genes, normalized to *actA*, are expressed relative to those obtained in −Fe. **B.** Protein quantification by Western blot analysis. Samples were obtained from the indicated strains grown as in **(A)**. Left panels: Representative Western blot analysis showing SidF and SidH protein levels. α-Tubulin was used as loading control. Right panels: Densitometric protein quantification. Protein levels were normalized to Tubuline and expressed relative to those in −Fe. Bars represent standard deviations from two independent biological experiments with two technical replicates each.

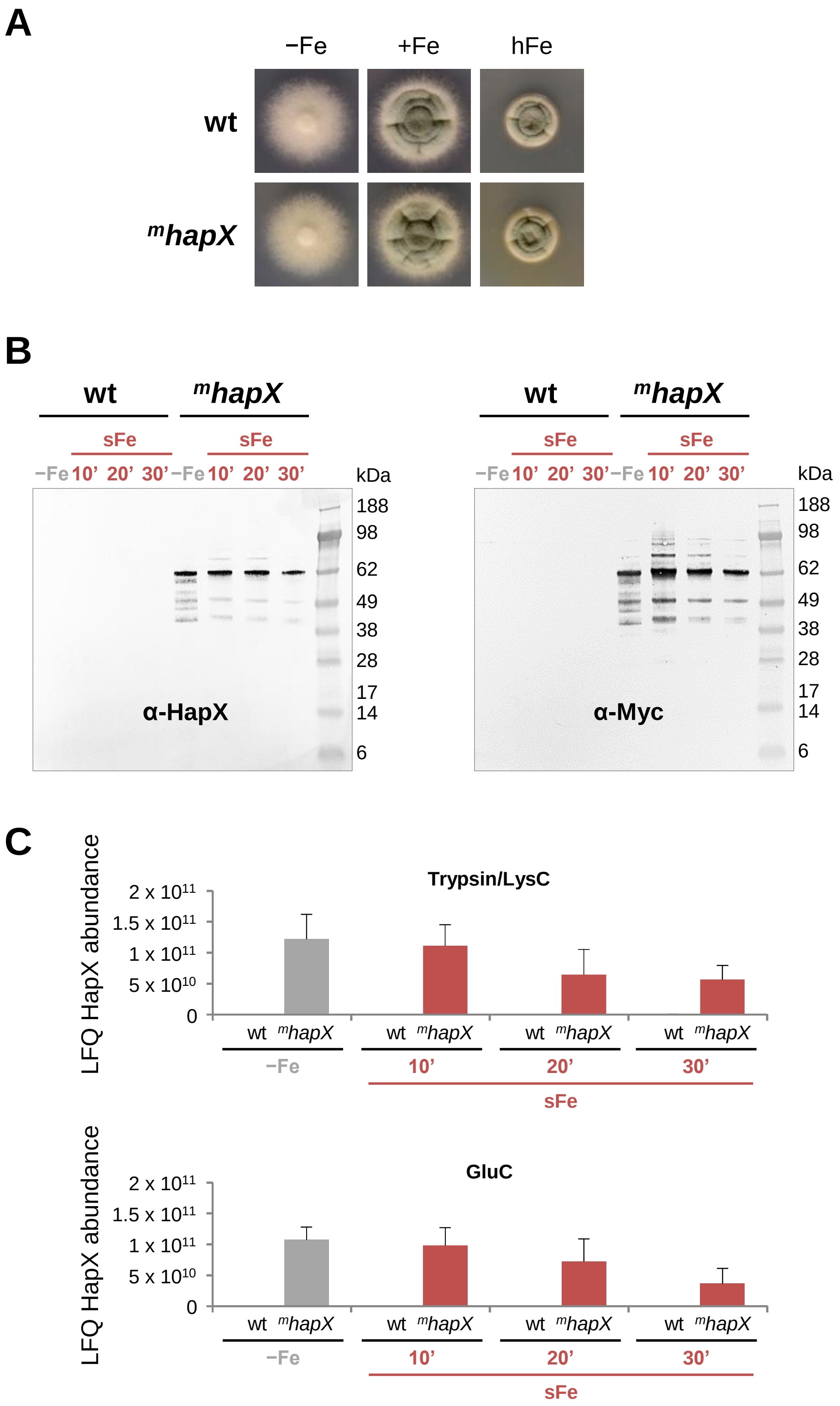

Figure S3. Effective enrichment of ^{M}HapX validated by Western blot and nLC-MS/MS analysis. A. Growth of the indicated strains on solid AMM −Fe (iron depletion), +Fe (30 µM $FeSO_4$), and hFe (10 mM $FeSO_4$). Plates were incubated for 2 d at 37ºC **B-C.** Samples were obtained from the wild-type and ^{m}*hapX* strains grown at 37ºC in AMM −Fe and then supplemented with 10 µM $FeSO_4$ for 10, 20 and 30 min (sFe) and crude extracts were subjected to Myc-Trap affinity purification. Eluates were subjected to Western blot analysis showing HapX levels with α-HapX and α-Myc antibodies **(B)**, or digested with Trypsin/LysC or GluC proteases for Label-Free Quantification (LFQ) abundances of HapX **(C)**. Bars represent standard deviations from three independent biological experiments.

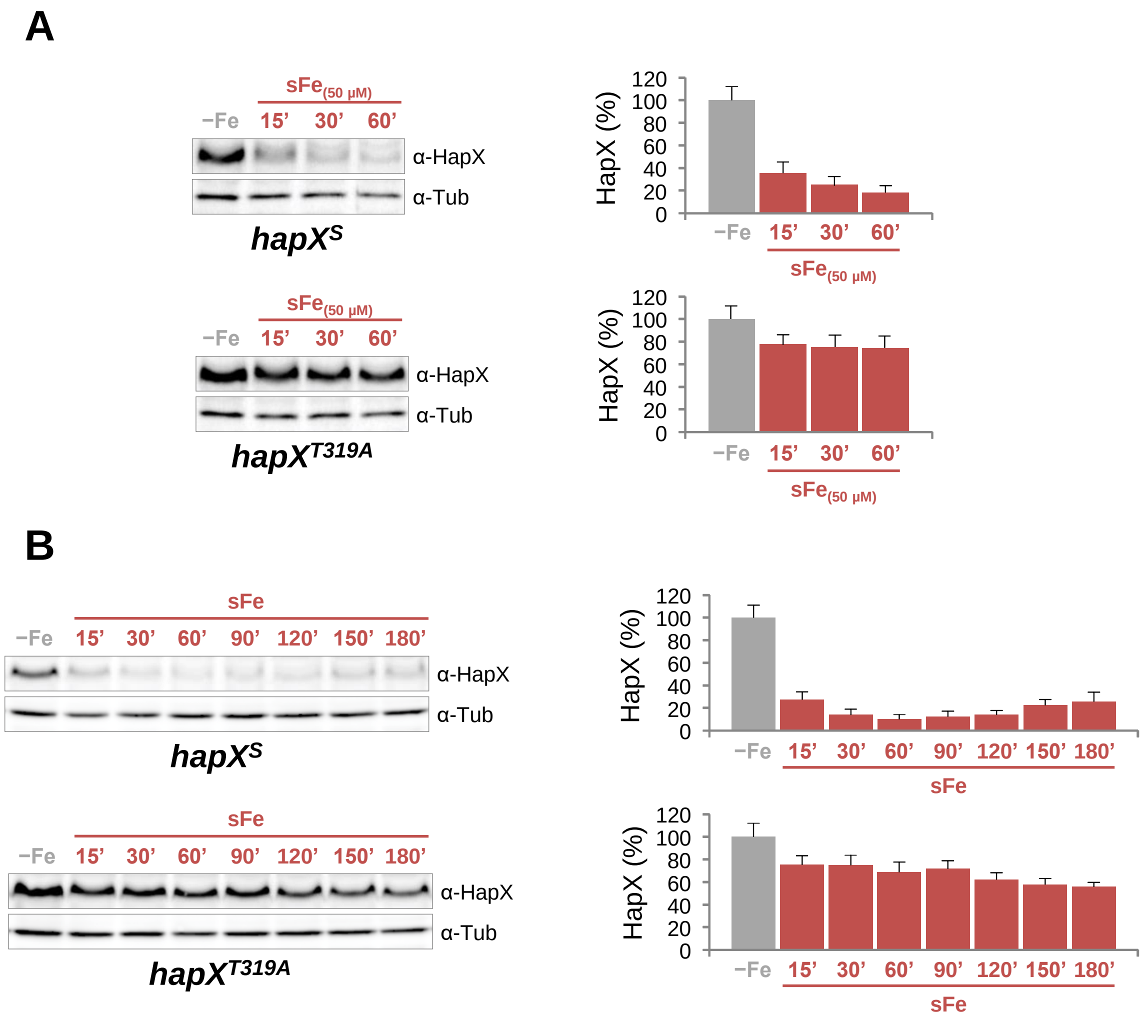

Figure S4. Quantification of HapX in *hapX^s* and *hapX^T319A* under different sFe conditions.
A-B. HapX quantification by Western blot analysis. Samples were obtained from the indicated strains grown at 37ºC in liquid AMM sFe (20 h in iron depletion and then supplemented with 50 μM $FeSO_4$ for 15, 30 and 60 min) **(A)**; or grown at 37ºC in AMM sFe (20 h in iron depletion and then supplemented with 10 μM $FeSO_4$ for the indicated time periods) **(B)**. Left panels: Representative Western blot analysis showing HapX protein levels. α-Tubulin was used as loading control. Right panels: Densitometric protein quantification. HapX protein levels were normalized to Tubuline and expressed relative to those in −Fe. Bars represent standard deviations from two independent biological experiments with two technical replicates each.

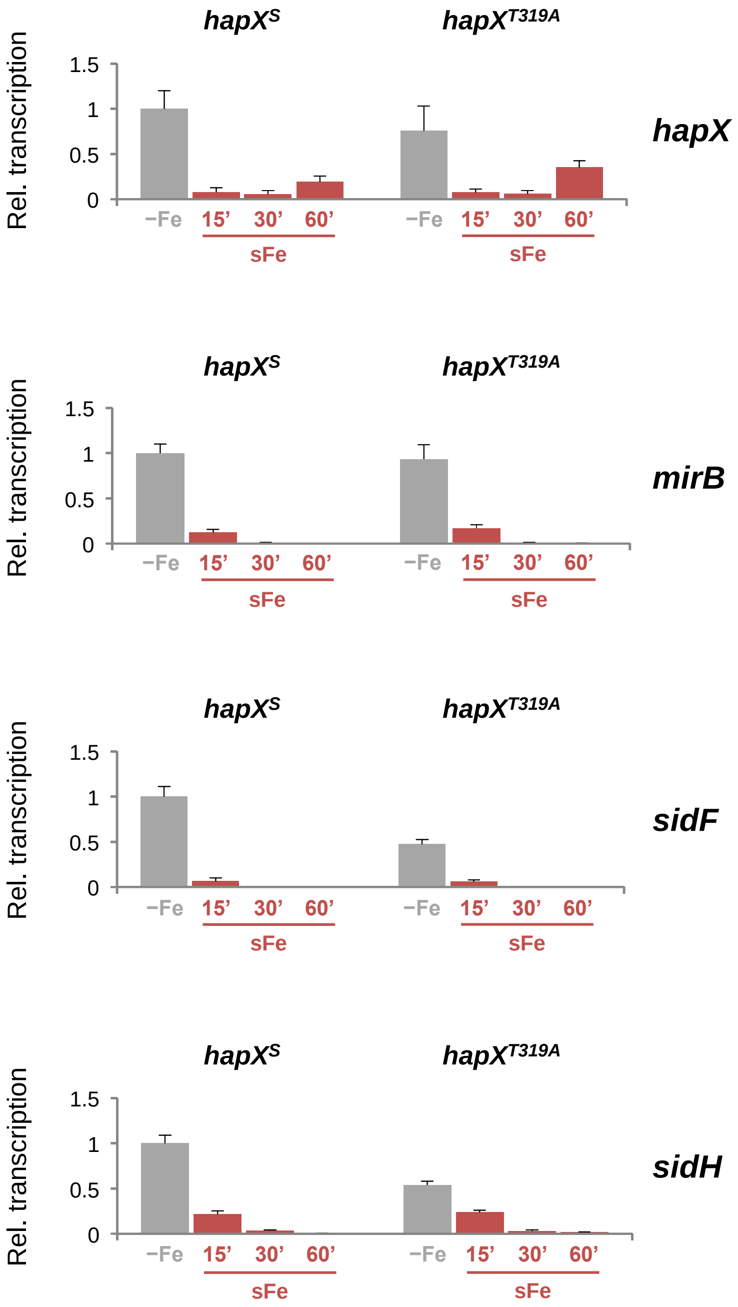

Figure S5. Transcription of iron-repressed genes.
Quantitative real-time reverse transcription RT-qPCR. Samples were obtained from the indicated strains grown as in Figure 2B. Transcript levels of the indicated genes, normalized to *actA*, are expressed relative to those obtained in −Fe. Bars represent standard deviations from two independent biological experiments with two technical replicates each.

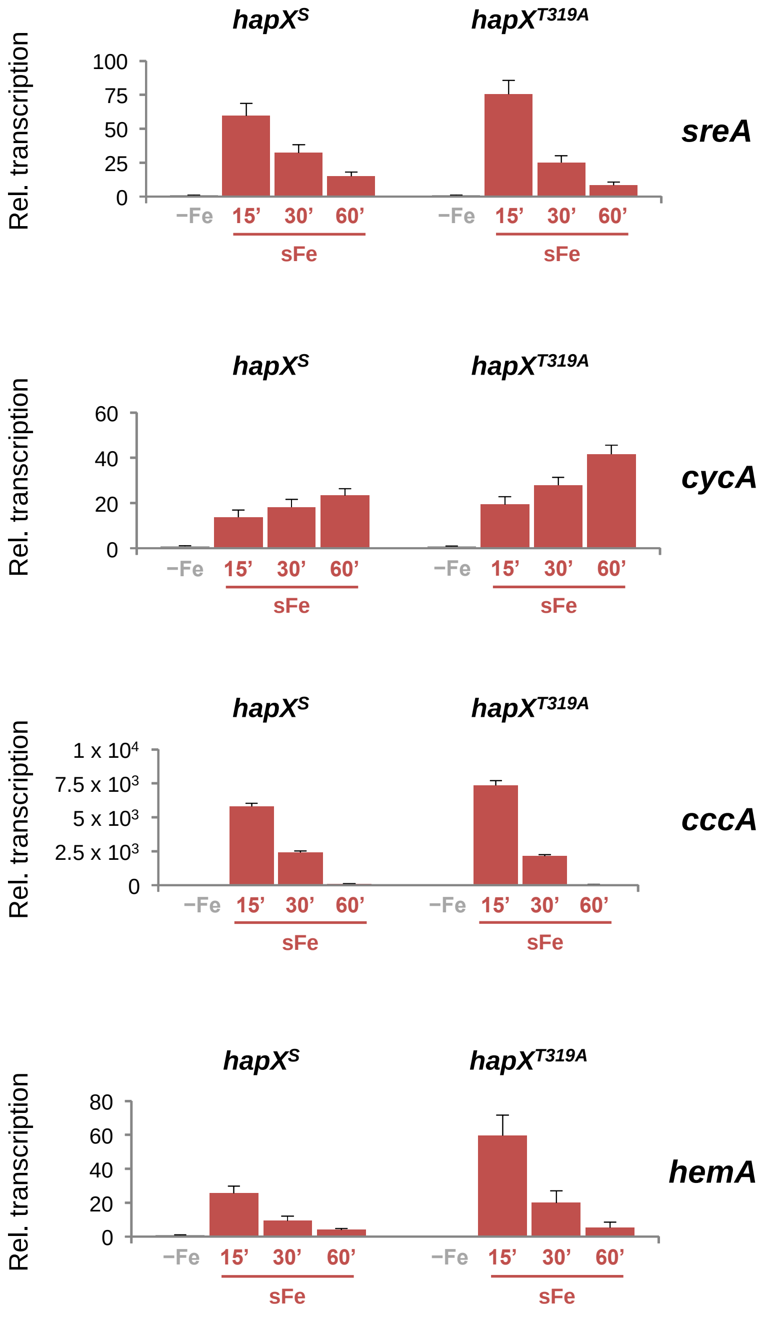

Figure S6. Transcription of iron-induced genes.

Quantitative real-time reverse transcription RT-qPCR. Samples were obtained from the indicated strains grown under sFe conditions as in Figure 2B. Transcript levels of the indicated genes, normalized to *actA*, are expressed relative to those obtained in −Fe. Bars represent standard deviations from two independent biological experiments with two technical replicates each.

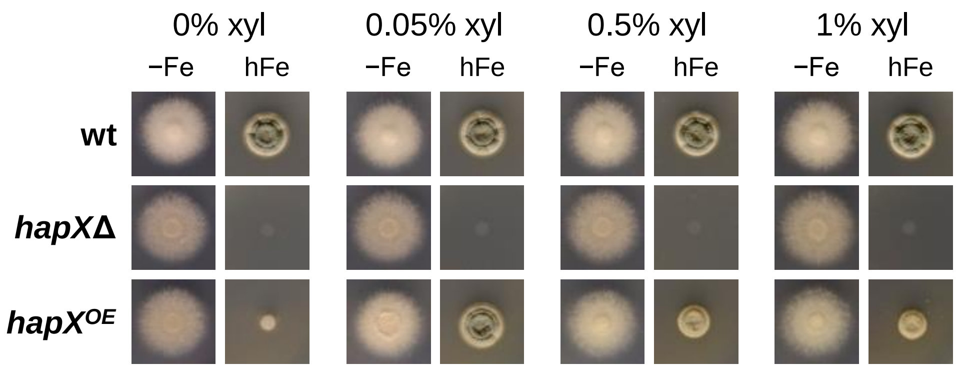

Figure S7. Effect of *hapX* overexpression on −Fe and hFe colony growth.
Growth of the indicated strains on solid AMM −Fe and hFe (10 mM $FeSO_4$) with the indicated concentration of xylose (%). Plates were incubated for 2 d at 37ºC.

```
                                                    ▼GluC
          A.fumigatus Af293 (56) STVRFLFDGTRVRPEDTPDSLDMADGDTLEVHQEQIGG------
                                      ▲Trypsin
         A.fumigatus A1163 (56) STVRFLFDGTRVRPEDTPDSLDMADGDTLEVHQEQIGG------
         A.fischeri NRRL 181 (54) STVRFLFDGTRVRPEDTPDSLDMADGDTLEVHQEQIGG------
        A.lentulus IFM 54703 (54) STVRFLFDGTRVRPEDTPDSLDMADGDTLEVHQEQIGG------
          A.clavatus NRRL 1 (54) STVRFLFDGTRVRPEDTPDTLDMSDGDTLEVHQEQIGG------
   A.thermomutatus HMR AF 39 (54) STVRFLFDGTRVRPEDTPDTLEMADGDTLEVHQEQIGG------
   A.novofumigatus IBT 16806 (88) STVRFLFDGTRVRPEDTPDSLDMADGDTLEVHQEQIGG------
          A.terreus NIH2624 (54) STVRFLFDGTRVRPEDTPDTLDMQDGDTLEVHQEQIGGGCL---
  A.brasiliensis CBS 101740 (51) STVRFLFDGTRVRPEDTPDTLEMADGDTLEVHQEQIGG------
     A.aculeatus ATCC 16872 (57) STVRFLFDGTRVRPEDTPDTLEMADGDTLEVHQEQIGG------
    A.campestris IBT 28561 (55) STVRFLFDGTRVRPEDTPDTLEMADGDTLEVHQEQIGG------
    A.carbonarius ITEM 5010 (55) STVRFLFDGTRVRPEDTPDTLEMTDGDTLEVHQEQIGG------
         A.flavus NRRL3357 (55) STVRFLFDGTRVRPEDTPDTLEMADGDTLEVHQEQIGG------
        A.glaucus CBS 516.65 (58) STVRFLFDGTRVRPEDTPDTLEMADGDTLEVHQEQIGG------
         A.wentii DTO 134E9 (45) STVRFLFDGTRVRPEDTPDTLEMADGDTLEVHQEQIGG------
A.ochraceoroseus IBT 24754 (54) STVRFLFDGTRVRPEDTPDTLDMADGDTLEVHQEQIGG------
        A.sydowii CBS 593.65 (55) STVRFLFDGTRVRPEDTPDTLDMADGDTLEVHQEQIGG------
     A.versicolor CBS 583.65 (55) STVRFLFDGTRVRPEDTPETLDMADGDTLEVHQEQIGG------
         A.nidulans FGSC A4 (55) STVRFLFDGTRVRPEDTPDTLDMADGDTLEVHQEQIGGGL----
     A.luchuensis CBS 106.47 (51) STVRFLFDGTRVRPEDTPDTLEMADGDTLEVHQEQIGG------
       A.luchuensis IFO 4308 (51) STVRFLFDGTRVRPEDTPDTLEMADGDTLEVHQEQIGG------
          A.niger ATCC 1015 (51) STVRFLFDGTRVRPEDTPDTLEMADGDTLEVHQEQIGG------
         A.niger ATCC 13496 (51) STVRFLFDGTRVRPEDTPDTLEMADGDTLEVHQEQIGG------
    A.niger N402 (ATCC 64974) (51) STVRFLFDGTRVRPEDTPDTLEMADGDTLEVHQEQIGG------
    A.tubingensis CBS 134.48 (51) STVRFLFDGTRVRPEDTPDTLEMADGDTLEVHQEQIGG------
          S.cerevisiae Smt3 (61) DSLRFLYDGIRIQADQTPEDLDMEDNDIIEAHREQIGGATY---
              S.pombe Pmt3 (74) NSLRFLVDGERIRPDQTPAELDMEDGDQIEAVLEQLGGCTHLCL
                  Consensus      STVRFLFDGTRVRPEDTPDTLEMADGDTLEVHQEQIGG
```

Figure S8. Amino acid sequence alignment of the C-terminal region of SumO orthologs from various *Aspergillus* species.

Sequences included represent all *A. fumigatus* SumO orthologs that were deposited in the publicly accessible database FungiDB (https://fungidb.org). Each sequence is preceded by its source organism and the strain ID. For comparison, SumO sequences from *Saccharomyces cerevisiae* and *Schizosaccharomyces pombe* are given. Identical residues are marked in yellow, residues conserved in 50% of the sequences are shaded in light blue and blocks of similar residues are marked in green. Red and black triangles mark the closest C-terminal GluC and trypsin P1 cleavage positions in *A. fumigatus* SumO, which after GluC digestion leaves a small residue of 4 amino acids (QIGG) on the modified lysine residue, while a large 27 amino acid residue signature (VRPEDTPDSLDMADGDTLEVHQEQIGG) remains after trypsin digestion (not detectable in LC-MS/MS analysis). Alignments were performed with AlignX (Vector NTI Advance 11).

Table S1. Strains used in this study

**Name**	**Genotype**	**Reference**
wt (AfS77)	*akuA*Δ	[55]
^{v}*sidF*	Afs77; *venus:sidF::hyg*	This work
^{v}*sidH*	Afs77; *venus:sidH::hyg*	This work
^{m}*hapX*	Afs77; *myc:hapX::hyg*	This work
*hapX*Δ	Afs77; *hapX*Δ*::ptrA*	[16]
*fbx22*Δ	Afs77; *fbx22*Δ*::hyg*	This work
*sumO*Δ	Afs77; *sumO*Δ*::hyg*	This work
$hapX^{s}$	Afs77; *hapX:Stag::hyg*	[16]
$hapX^{K161R}$	Afs77; $hapX^{K161R}$*:Stag::hyg*	This work
$hapX^{K242R}$	Afs77; $hapX^{K242R}$*:Stag::hyg*	This work
$hapX^{T319A}$	Afs77; $hapX^{T319A}$*:Stag::hyg*	This work
$hapX^{K161R\text{-}K242R}$	Afs77; $hapX^{K161R\text{-}K242R}$*:Stag::hyg*	This work
$hapX^{K161R\text{-}T319}$	Afs77; $hapX^{K161R\text{-}T319A}$*:Stag::hyg*	This work
$hapX^{K242R\text{-}T319A}$	Afs77; $hapX^{K242R\text{-}T319A}$*:Stag::hyg*	This work
$hapX^{OE}$	Afs77; *PxylP:hapX:Stag::hyg*	This work

55. Hartmann, T.; Dumig, M.; Jaber, B. M.; Szewczyk, E.; Olbermann, P.; Morschhauser, J.; Krappmann, S., Validation of a self-excising marker in the human pathogen *Aspergillus fumigatus* by employing the beta-rec/six site-specific recombination system. *Appl Environ Microbiol* **2010,** 76, (18), 6313-7.

16. Gsaller, F.; Hortschansky, P.; Beattie, S. R.; Klammer, V.; Tuppatsch, K.; Lechner, B. E.; Rietzschel, N.; Werner, E. R.; Vogan, A. A.; Chung, D.; Muhlenhoff, U.; Kato, M.; Cramer, R. A.; Brakhage, A. A.; Haas, H., The Janus transcription factor HapX controls fungal adaptation to both iron starvation and iron excess. *The EMBO journal* **2014,** 33, (19), 2261-76.
